# Aqueous photo-RAFT polymerization under ambient conditions: synthesis of protein–polymer hybrids in open air†

**DOI:** 10.1039/d4sc01409j

**Published:** 2024-05-24

**Authors:** Arman Moini Jazani, Hironobu Murata, Martin Cvek, Anna Lewandowska-Andralojc, Roksana Bernat, Kriti Kapil, Xiaolei Hu, Ferdinando De Luca Bossa, Grzegorz Szczepaniak, Krzysztof Matyjaszewski

**Affiliations:** a Department of Chemistry, Carnegie Mellon University 4400 Fifth Avenue Pittsburgh PA 15213 USA km3b@andrew.cmu.edu; b Centre of Polymer Systems, Tomas Bata University in Zlin Trida T. Bati 5678 76001 Zlin Czech Republic; c Faculty of Chemistry, Adam Mickiewicz University Uniwersytetu Poznanskiego 8 61-614 Poznan Poland; d Center for Advanced Technology, Adam Mickiewicz University Uniwersytetu Poznanskiego 10 61-614 Poznan Poland; e Institute of Materials Engineering, University of Silesia 75 Pulku Piechoty 1A 41-500 Chorzow Poland; f Faculty of Chemistry, University of Warsaw Pasteura 1 02-093 Warsaw Poland

## Abstract

A photoinduced reversible addition-fragmentation chain-transfer (photo-RAFT) polymerization technique in the presence of sodium pyruvate (SP) and pyruvic acid derivatives was developed. Depending on the wavelength of light used, SP acted as a biocompatible photoinitiator or promoter for polymerization, allowing rapid open-to-air polymerization in aqueous media. Under UV irradiation (370 nm), SP decomposes to generate CO_2_ and radicals, initiating polymerization. Under blue (450 nm) or green (525 nm) irradiation, SP enhances the polymerization rate *via* interaction with the excited state RAFT agent. This method enabled the polymerization of a range of hydrophilic monomers in reaction volumes up to 250 mL, eliminating the need to remove radical inhibitors from the monomers. In addition, photo-RAFT polymerization using SP allowed for the facile synthesis of protein–polymer hybrids in short reaction times (<1 h), low organic content (≤16%), and without rigorous deoxygenation and the use of transition metal photocatalysts. Enzymatic studies of a model protein (chymotrypsin) showed that despite a significant loss of protein activity after conjugation with RAFT chain transfer agents, the grafting polymers from proteins resulted in a 3–4-fold recovery of protein activity.

## Abstract 2

A photoinduced reversible addition-fragmentation chain-transfer (photo-RAFT) polymerization technique in the presence of sodium pyruvate (SP) and pyruvic acid derivatives was developed.

## Graphical abstract

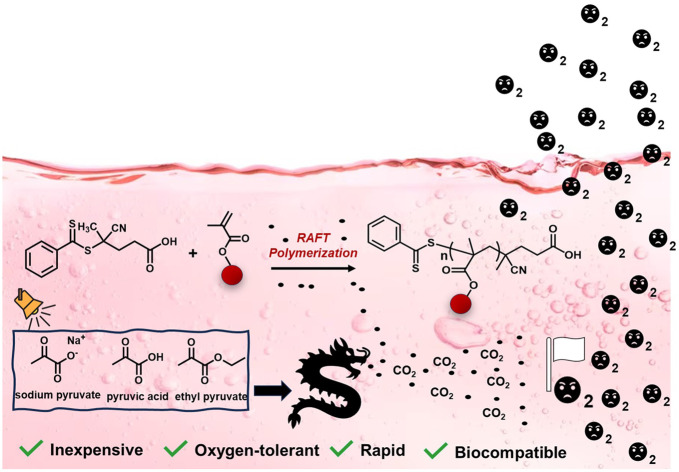

## Introduction

Reversible-deactivation radical polymerization (RDRP), also known as controlled radical polymerization (CRP), has emerged as a powerful synthetic tool for preparing polymers with complex architectures, narrow molecular weight distributions, tunable chain end functionality, and predetermined molecular weights.^1–4^ Reversible addition-fragmentation chain-transfer (RAFT) polymerization and atom transfer radical polymerization (ATRP) are among the most widely used and studied RDRP methods for a variety of biomedical applications such as drug delivery, immunotherapy, tissue engineering, and medical imaging.^5,6^ Both ATRP and RAFT polymerization can be performed in water,^7–10^ opening new avenues for the synthesis of protein-, DNA-, RNA- or cell-polymer bioconjugates.^11–15^

Proteins are at the forefront of new therapeutic discoveries for many diseases, but *in vivo* application has been largely constrained by their poor blood circulation, instability to proteolytic degradation, and ability to elicit an immune response.^16,17^ Weaving proteins with polymeric shells to form protein–polymer hybrids (PPHs) offers a unique opportunity to improve the pharmacokinetic properties of proteins while retaining them sufficiently active *in vivo*.^18–21^ Indeed, there are numerous examples of FDA-approved poly(ethylene glycol) (PEG)-based PPHs in clinical use and evaluation.^22,23^ PPHs are typically synthesized by the “grafting to” approach, where pre-synthesized polymers are coupled to the proteins *via* reactive handles, such as lysine or cysteine residues available on the protein surface. Nevertheless, “grafting to” has disadvantages such as purification difficulties and poor conjugation efficiency.^11,24,25^ In an alternative approach, termed “grafting from”, polymers are progressively grown from the initiators/chain transfer agents anchored to the protein surface *via* RDRP, which facilitates their purification and conjugation.^26–29^

**Fig. 1:**
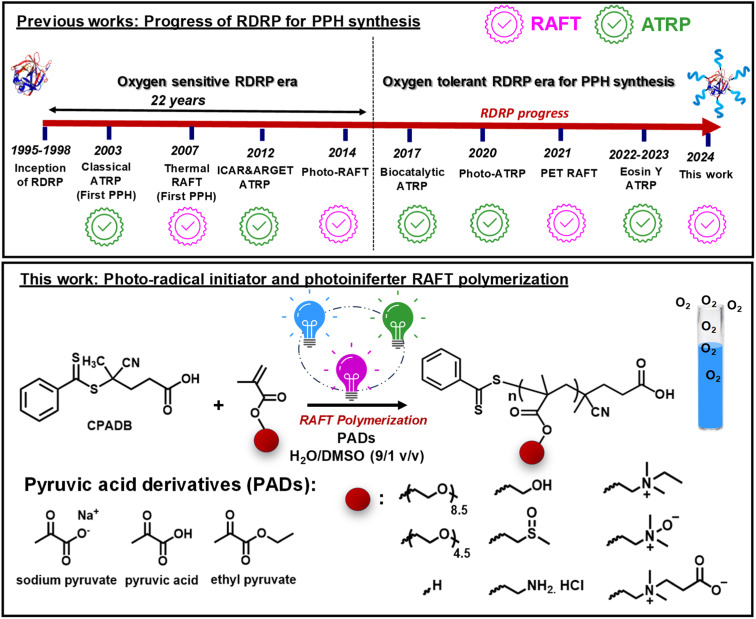
Development of aqueous ATRP and RAFT polymerization for PPH synthesis.

While both RAFT polymerization and ATRP have been extensively studied to graft from proteins,^30–37^ little effort has been devoted to introducing oxygen tolerance into the bioconjugate synthesis (Fig. 1).^38–42^ This is particularly important because conventional degassing methods (*i.e.*, inert-gas sparging or freeze–pump–thaw degassing) can lead to a loss of enzymatic activity and protein denaturation.^43–46^ In addition, oxygen tolerance in PPH synthesis can improve the reproducibility, cost-effectiveness, practicality, and user-friendliness of PPH synthesis for non-experts. Inspired by the ever-growing interest in performing RDRP under ambient conditions,^47–49^ biocatalytic ATRP was developed for PPH synthesis using glucose oxidase (GOx) as the degassing enzyme.^39^ Nevertheless, the use of GOx for bioconjugate synthesis is largely hampered by the difficulty of removing it from the final biohybrids. Oxygen-tolerant PPH synthesis *via* UV-induced ATRP and *in situ* disproportionation of the Cu(i) complex was also demonstrated.^40,41^ Finally, our group recently reported oxygen-tolerant synthesis of acrylate/methacrylate-based linear and hyperbranched PPHs *via* green-light-driven ATRP enabled by photoredox/copper dual catalysis (Fig. 1).^50–52^

PPH synthesis *via* oxygen-tolerant RAFT polymerization is relatively less explored. An important advancement was the development of the photoinduced electron/energy transfer process (PET-RAFT polymerization), in which photoredox catalysts are used in conjunction with light irradiation to trigger RAFT polymerization.^53–58^ PET-RAFT polymerization has gained widespread popularity, which can be explained by its oxygen tolerance and ability to initiate polymerization without external initiators, rendering it the only oxygen-tolerant RAFT polymerization method used for biohybrid synthesis so far.^59–62^ However, PET-RAFT polymerization inevitably leads to contamination with highly colored and sometimes toxic photocatalysts,^63^ which can strongly bind to biomolecules (*i.e.*, DNA and proteins), due to their conjugated and charged properties, thereby limiting their applications in biomolecule synthesis. Furthermore, PET-RAFT typically requires the use of transition metals, organic electron donors (*i.e.*, triethanolamine), or singlet oxygen scavengers (*e.g.*, DMSO or thioethers) to impart the system with sufficient oxygen tolerance.^64^

While the toolkit of oxygen-tolerant RAFT polymerization in water continues to expand,^65–68^ many of these methods have only shown limited tolerance to oxygen, enabling polymerization only in closed reaction vessels, without headspace and without stirring (Table S2 in the ESI† for examples of oxygen-tolerant RAFT polymerization in water). RAFT polymerization in open-to-air flasks remarkably increases the practicality of the experimental procedures; although, it is more difficult due to the continuous diffusion of oxygen during polymerization. To date, examples of aqueous RAFT polymerization that can be performed in open reaction vessels are mostly accessible *via* enzyme degassing methods^69^ or thermal radical initiator-based approaches (*i.e.*, VA-044).^70,71^ However, the thermal radical initiator-based aqueous RAFT polymerization for biohybrid synthesis comes with additional challenges. Mostly, denaturation and loss of activity of proteins are common at higher temperatures.

We introduce herein a versatile method for aqueous RAFT polymerization under ambient conditions, employing radicals generated by photodecomposition of highly biocompatible pyruvic acid derivatives (PADs). The use of PADs in RDRP originated from sodium pyruvate (SP), a common component of cell culture media, as a hydrogen peroxide (H_2_O_2_) scavenger in oxygen-tolerant ATRP mediated by GOx.^38^ Later, we demonstrated that SP undergoes homolytic photodecarboxylation and reduces copper(ii) or iron(iii), enabling open-to-air photo-ATRP of methacrylates and methacrylamides.^72,73^ Inspired by these studies and recent studies, where PADs were used as highly biocompatible and non-aromatic radical initiators,^74^ we applied SP to induce RAFT polymerization in water. Particularly, the ability of SP to confer RAFT polymerization with oxygen tolerance was of paramount importance due to its application in PPH synthesis.

## Results and discussion

### Initial optimization of photo-RAFT polymerization

RAFT polymerization of oligo(ethylene oxide) monomethyl ether methacrylate (OEOMA_500_, average *M*_n_ = 500, 8.5 average number of repeating units) was conducted using 4-cyano-4-(phenylcarbonothioylthio)pentanoic acid (CPADB) as the RAFT agent and SP as the photoinitiator, in a water/DMSO (9/1 v/v) mixture. The molar ratio of [OEOMA_500_]_0_/[CPADB]_0_/[SP]_0_ was 100/1/85 (Table 1 and Fig. S1† for SEC traces). Polymerizations were performed in completely open-to-air glass vials without prior deoxygenation. After 1 h of UV irradiation (370 nm, 6.5 mW cm^−2^) at a monomer concentration of 800 mM, a polymer with low dispersity (*Đ* = 1.26) and good agreement between theoretical molar mass (*M*_n,th_ = 40 300) and absolute molar mass (*M*_n,abs_ = 41 300) was obtained (entry 1, Table 1). Control experiments without SP or UV irradiation resulted in negligible monomer conversion (<5%, entries 2 and 3, Table 1). The irradiation of the polymerization mixture without CPADB formed polymers with high dispersity (*Đ* = 4.20), demonstrating the photo-induced radical generation properties of SP.

**Table 1:** Photo-RAFT polymerization of OEOMA_500_^a^

Entry	PAD	Light (nm)	[OEOMA_500_]_0_ (mM)	Time (h)	Conv.^b^ (%)	*M*_n,th_	*M*_n,app_ c	*M*_n,abs_ d	*Đ* c
1	SP	370	800	1	80	40 300	33 200	41 300	1.26
2	—	370	800	1	<5	—	—	—	—
3	SP	—	800	1	<5	—	—	—	—
4	SP	370	300	1	83	41 800	32 000	51 600	1.19
5^e^	SP	370	300	1	91	45 800	35 900	46 200	1.18
6	SP	450	800	7	91	45 800	27 000	48 900	1.24
7	—	450	800	7	71	35 800	23 700	41 300	1.22
8	SP	525	800	7	83	41 800	26 000	47 300	1.23
9	—	525	800	7	63	31 800	20 000	35 400	1.26
10	SP	450/525	300	7	<5	—	—	—	—
11	EP	370	300	2	99	49 800	29 100	55 300	1.26
12	PA	370	300	1	61	30 800	22 400	34 500	1.24

a Reaction conditions: [OEOMA_500_]_0_/[CPADB]_0_/[PADs]_0_ = 100/1/85 in a water/DMSO (9/1 v/v) mixture, irradiated with Kessil LEDs 370 nm (6.5 mW cm^−2^), 450 nm (21.5 mW cm^−2^), or 525 nm (20.0 mW cm^−2^) in an open-to-air glass vial.

b Monomer conversion was determined by ^1^H-NMR spectroscopy.

c Molecular weight (*M*_n,app_) and dispersity (*Đ*) were determined by SEC analysis (DMF as the eluent) calibrated with poly(methyl methacrylate) standards.

d Absolute molecular weight (*M*_n,abs_) was determined using d*n*/d*c* values of POEOMA_500_ = 0.05 using a SEC equipped with a MALS detector; dispersity (*Đ*) values were recorded from refractive index traces.

e Without DMSO.

Next, RAFT polymerization at a lower monomer concentration ([OEOMA_500_]_0_ = 300 mM) was carried out to improve the system compatibility for biomolecule grafting. Under UV irradiation, the RAFT polymerization proceeded at a comparable rate (conv. = 83%, 1 h) and showed better polymerization control (*Đ* = 1.19, entry 4, Table 1). DMSO was initially used to improve the solubility of CPADB. However, even trace amounts of DMSO can disrupt the secondary structure of proteins.^75,76^ Therefore, polymerization was performed without DMSO, which did not adversely affect the polymerization rate or control (*M*_n,abs_ = 46 200, *Đ* = 1.18, entry 5, Table 1). This shows that RAFT polymerization with SP eliminates the need for the use of DMSO as an oxygen scavenger, unlike PET-RAFT polymerization, where DMSO is typically required to scavenge the singlet oxygens.^77,78^ SP effectively scavenges oxygen through reactions with radicals generated from its photodecomposition (*vide infra*). Notably, polymerization at lower SP concentrations ([SP]_0_ ≤ 32 mM) did not afford high conversions within 1 h (Fig. S2†). Due to the possibility of UV-induced protein damage, particularly with prolonged UV irradiation, all subsequent experiments were conducted at higher SP concentrations (128 or 256 mM), which resulted in rapid polymerization.

To evaluate the effectiveness of our approach at longer wavelengths, polymerizations using blue (450 nm, 21.5 mW cm^−2^) and green (525 nm, 20.0 mW cm^−2^) light irradiation were performed (Fig. 4A). In both cases, controlled polymerization occurred, yielding a high monomer conversion (>80%, entries 6 and 8, Table 1). Interestingly, RAFT polymerization proceeded in the open vials even without SP, albeit with lower conversions (71% for blue and 63% for green light, entries 7 and 9, Table 1). However, at a lower monomer concentration ([OEOMA_500_]_0_ = 300 mM, entry 10, Table 1), polymerization did not occur with either blue or green light irradiation, regardless of the presence of SP. This suggests that the photo-iniferter (PI) RAFT mechanism contributes to the activation of CPADB under lower energy light, allowing polymerization even under aerobic conditions (*vide infra*).^79–81^

Finally, we investigated the applicability of our approach with pyruvic acid (PA) and other PADs, including ethyl pyruvate (EP), sodium oxalate (SO), and oxalic acid (OA). Only EP and PA afforded high monomer conversions (99% and 61%, respectively), while no conversion was observed with SO and OA, which could be attributed to their limited solubility in water. Polymerization with EP required a longer polymerization time (2 h) to reach high conversions compared to SP and PA, suggesting slower radical generations. This was attributed to the lack of hydrogen-bonding of carboxylic acid functional groups in EP since the photochemistry of PA is related to the formation of hydrogen-bonding dimers.^82^

### Kinetics of photo-RAFT polymerization

**Fig. 2:**
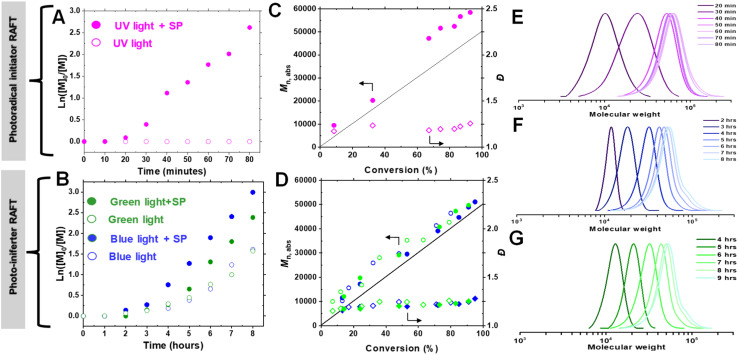
Kinetics of photo-RAFT polymerization of OEOMA_500_. Polymerization under irradiation with UV light (A), green or blue light (B). Evolution of absolute molecular weight (*M*_n,abs_) and dispersity (*Đ*) for polymerization under irradiation with UV light (C) or green or blue light (D). SEC trace evolution for polymerization in the presence of SP under irradiation with UV light (E), blue light (F), and green light (G). Polymerizations were carried out in an open-to-air vessel in a water/DMSO (9/1 v/v) mixture. [OEOMA_500_]_0_/[CPADB]_0_/[SP]_0_ = 100/1/85, [OEOMA_500_]_0_ = 300 mM for polymerization with UV light and [OEOMA_500_]_0_ = 800 mM for polymerization with green or blue light. Irradiation with Kessil UV (370 nm, 6.5 mW cm^−2^), blue (450 nm, 21.5 mW cm^−2^) and green (525 nm, 20.0 mW cm^−2^) light.

To elucidate the mechanism of photo-RAFT polymerization using SP, the effects of UV (370 nm, 6.5 mW cm^−2^), blue (450 nm, 21.5 mW cm^−2^), and green (525 nm, 20.0 mW cm^−2^) light on the polymerization kinetics were investigated (Fig. 2). As described earlier, in the absence of SP, no polymerization was observed upon UV irradiation. Conversely, in the presence of SP, monomer conversion reached >80% within 1 h after an induction period of ∼15 min (Fig. 2A). This observation suggests that the polymerization is driven by radicals generated from the photodecomposition of SP. The UV-vis spectrum of SP further supports the overlap of the SP absorption band with the emission of UV light (Fig. 4B).

Upon exposure to blue or green light, RAFT polymerization was initiated even in the absence of SP, resulting in a slow polymerization that reached over 60% monomer conversion after 7 h (Fig. 2B). Since the emission spectra of green and blue light do not overlap with the π → π* absorption of CPADB, the observed polymerization was attributed to the weak absorption of light by the forbidden n → π* transition of CPADB (Fig. 4B). This photoexcitation led to the homolytic cleavage of a C–S bond and the generation of radicals *via* the PI mechanism.^79,80,83,84^ Surprisingly, PI-RAFT polymerization with CPADB exhibited remarkable oxygen tolerance, and reached high conversions even in vials without caps.^85^ Another intriguing observation was the suppression of the induction period and increase in the rate of polymerization in the presence of SP, which was enhanced nearly 2-fold (*k*^app^_p_ = 0.91 × 10^−2^ min^−1^ and 0.78 × 10^−2^ min^−1^ with SP *vs.* 0.51 × 10^−2^ min^−1^ 0.46 × 10^−2^ min^−1^ without SP for blue and green light, respectively). This could be attributed to interaction of SP with the CPADB in the excited state (*vide infra*). This finding aligns with previous reports demonstrating the ability of additives, such as triethylamine, to accelerate PI-RAFT polymerization.^86–88^

In all cases, the absolute molecular weight (*M*_n,abs_) increases in reasonable agreement with the theoretical values (Fig. 2C and D), indicating that the formation of free polymer chains was not significant despite the relatively high concentration of SP. Although some radicals generated from SP are possibly quenched by O_2_, the remaining SP radicals should initiate new chains once all O_2_ is removed. The fraction of new chains was, however, insignificant, as evidenced by the excellent agreement of *M*_n,abs_ and *M*_n,th_. Additionally, the polymer dispersity remained low (*Đ* = 1.13–1.28) over time. Finally, the SEC traces were narrow and unimodal throughout the polymerization, demonstrating the livingness of the photo-RAFT polymerization under exposure to all three different light sources (Fig. 2E–G).

### Oxygen tolerance of photo-RAFT polymerization

**Fig. 3:**
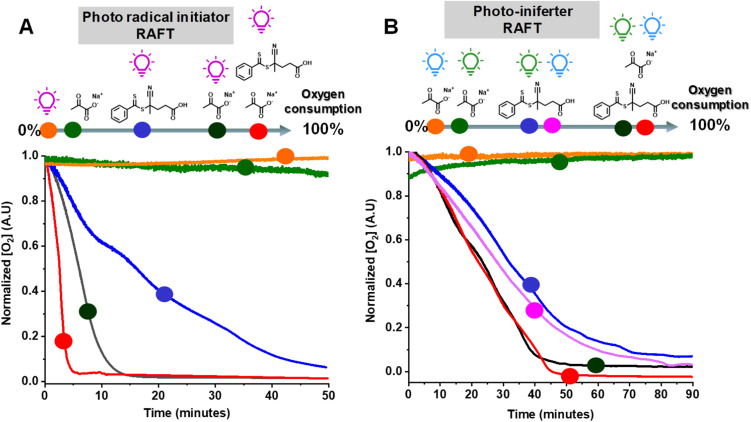
Oxygen concentration measurements under photo-radical initiator RAFT conditions (A), and PI RAFT conditions (B). Measurements were performed in an open-to-air vessel in a water/DMSO (9/1 v/v) mixture (without monomer). [CPADB]_0_/[SP]_0_ = 1/85, [SP]_0_ = 256 mM and [CPADB]_0_ = 3.0 mM. Irradiation with Kessil UV (370 nm, 6.5 mW cm^−2^), blue (450 nm, 21.5 mW cm^−2^) and green (525 nm, 20.0 mW cm^−2^) light.

We then examined the effect of various components on the rate of removal of dissolved oxygen in a water/DMSO solution (9/1 v/v) using an oxygen probe (Fig. 3A). Oxygen measurements were performed in an open vessel (1.5 mL vial). The oxygen concentration remained constant in the absence of UV irradiation (green circle) or SP (orange circle). However, the combination of SP and UV irradiation (black circle) depleted all oxygen within 15 min. Oxygen measurements in the presence of CPADB exposed to UV light (blue circle) indicated that oxygen removal was still possible under this condition, but at a significantly slower rate. The most remarkable oxygen scavenging was observed when all components (UV light, CPADB, and SP) were present, with complete oxygen depletion occurring within ≤5 min (red circle).

**Fig. 4:**
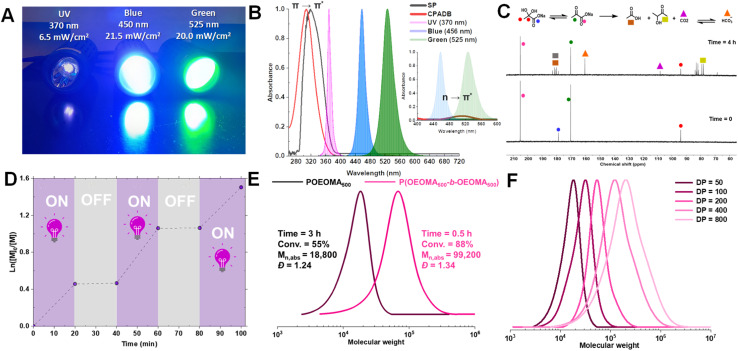
Digital image of Kessil LEDs used for polymerization (A); UV-vis spectra of SP (black) and CPADB (red) compared with the emission of Kessil UV, blue, and green light. The inset shows the n → π* transition of CPADB, which overlaps with the emission of green and blue LEDs (B); ^13^C-NMR of SP before irradiation (bottom) and after irradiation with UV (top) shows the appearance of new peaks assigned to the formation of HCO_3_^−^ and other photodecomposition products (*i.e*., acetoin and lactic acid) (C); temporal control of polymerization of photo-RAFT with UV irradiation and SP (D); *in situ* chain extension of OEOMA_500_ (DP = 150) from the POEOMA_500_ macroinitiator (DP = 50) (E); photo-RAFT polymerization of OEOMA_500_ at different target DPs (*i.e.*, 50, 100, 200, 400, and 800) (F).

The remarkable oxygen tolerance of our system can be attributed to the rapid photodecomposition of SP, which generated a continuous flux of radicals that reacted with oxygen to form unreactive peroxy radicals.^47,61,71^ To investigate the photodecomposition of SP, a solution of SP in water was exposed to UV light (370 nm, 26.3 mW cm^−2^) and subsequently analyzed by ^13^C-NMR (Fig. 4C) and ^1^H-NMR (Fig. S3†). As shown in Fig. 4C, SP was in equilibrium with sodium 2,2-dihydroxypropanoate in aqueous media. After 4 h of irradiation, new peaks corresponding to CO_2_ and HCO_3_^−^ were observed in the ^13^C-NMR spectrum. Additionally, other photodecomposition products of SP, including acetoin, acetic acid, and lactic acid were detected.^82,89^

PI-RAFT polymerization exhibited unprecedented oxygen tolerance, both with and without SP. The oxygen measurements revealed that the oxygen concentration remained unchanged under blue (orange circle) or green light irradiation (green circle) in the presence of SP alone (without CPADB), ruling out the possibility of its photodecomposition due to UV light contamination of the visible light sources. Conversely, CPADB caused a significant decrease in oxygen concentration after 90 min of irradiation with either green (blue circle) or blue light (purple circle). Consistent with the polymerization kinetics experiments, the addition of SP accelerated the rate of oxygen depletion in PI-RAFT polymerization (red and black circles).

### Mechanism of photo-RAFT polymerization

**Scheme 1:**
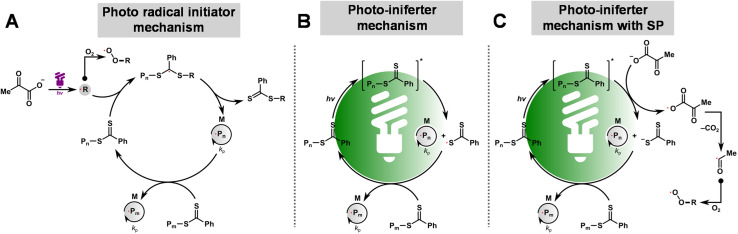
Proposed mechanism for photo-RAFT polymerization upon exposure to light of different wavelengths: the photo-radical initiator mechanism (A), PI mechanism (B), and PI mechanism with SP (C).

The combined kinetic results and oxygen measurements indicate that SP acts as a photo-initiator or promoter for RAFT polymerization, depending on the wavelength of light used. Under UV irradiation (370 nm), SP decomposes to form an acetyl radical, CO_2_, and other species (*i.e.*, acetic acid and acetoin; see Fig. 4C).^89^ The formed radicals initiate polymerization, controlled by a degenerative chain transfer process (Scheme 1A). In addition, the radicals generated from the photodecomposition of SP can react with oxygen to form unreactive peroxy radicals, providing oxygen tolerance to the system. Under green and blue light irradiation, the reaction proceeds by the PI mechanism in which CPADB is directly excited, leading to carbon–sulfur bond homolysis (Scheme 1B). In addition, electron transfer from SP to the excited CPADB can occur, resulting in the formation of the CPADB radical anion and the acyloxyl radical (Scheme 1C, see discussion in the ESI and Fig. S4–S8†). The CPADB radical anion then cleaves to form a carbon-centered radical and a CPADB anion. The acyloxyl radical decomposes to form an acetyl radical and CO_2_. The continuous generation of radicals in this process increases the polymerization rate and oxygen tolerance.

### Versatility of photo-RAFT polymerization

To evaluate the temporal control and the livingness of end chains, an on/off light study was conducted. The results demonstrated that the photo-RAFT polymerization could be effectively halted/restarted by switching on/off the light source (Fig. 4D). Moreover, the high end-group fidelity of the synthesized polymers was confirmed by both ^1^H-NMR and chain extension experiments. In ^1^H-NMR, the distinct chemical shifts of the dithioesters were clearly detected at 7.2–7.9 ppm (Fig. S9†). For the chain extension experiment, the POEOMA_500_ macro-RAFT agent was obtained (DP = 50, conv. = 55%, 3 h). The unpurified macro-RAFT agent aliquots were then used for chain extension with a new batch of OEOMA_500_ under UV irradiation (DP = 150, conv. = 88%, 0.5 h). The significant shift of the GPC traces towards high molecular weights, along with the absence of low molecular weight tailing, confirms that this method can be used to synthesize polymers with high chain end functionality (Fig. 4E). In addition, the SP-RAFT polymerization was used to synthesize POEOMA_500_ with a range of target DPs (50–800). However, control over the polymerization process diminished for higher target DPs (DP ≥ 400) (Table S3†), confirming some previous findings where photo-RAFT polymerization was unable to produce high-molecular-weight polymers with a narrow molecular-weight distribution.^50,90^ Polymerization at lower DP required a longer period of irradiation to reach high conversion.

### Scaling up SP-RAFT polymerization

**Fig. 5:**
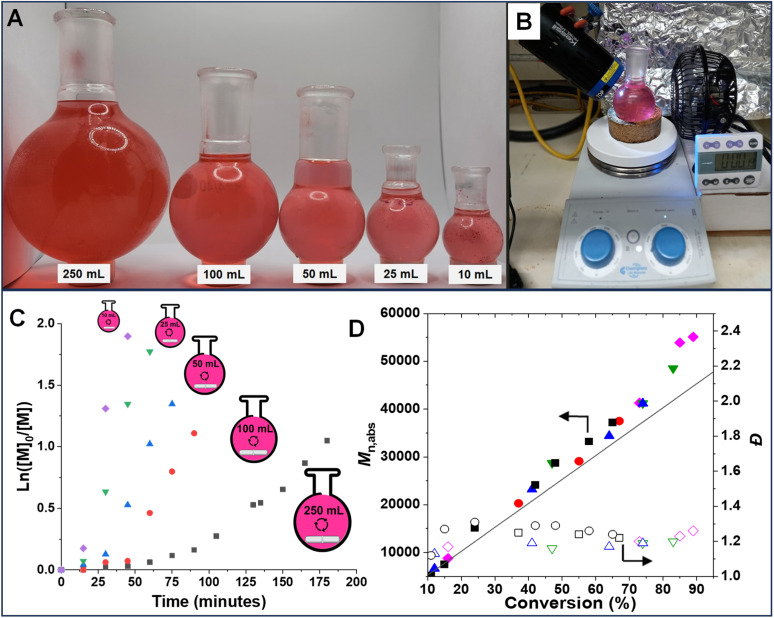
Scaling up of photo-RAFT polymerization of OEOMA_500_ with SP, under irradiation with UV light. Digital image of the polymerization set-up in open-to-air round bottom flasks (*i.e.*, 10, 25, 50, 100 and 250 mL) (A and B). Kinetics of polymerization of OEOMA_500_ by large-scale photo-RAFT polymerization. Semilogarithmic kinetic plots (C), evolution of absolute molecular weight (*M*_n,abs_) and dispersity (*Đ*) for polymerization under irradiation with UV light (D).

To demonstrate the versatility and effectiveness of this technique on a larger scale, even in the presence of the inhibitor (monomethyl ether hydroquinone, 900 ppm), polymerization at five different scales (10, 25, 50, 100 and 250 mL) was performed. This was achieved by using a simple polymerization setup (Fig. 5B) in open-to-air round bottom flasks (Fig. 5A). Stirring the polymerization mixture allowed for continuous oxygen diffusion into the system. The photo-RAFT polymerization exhibited characteristics of living polymerization at all scales, as evidenced by a linear increase in molecular weight and close agreement between *M*_n,th_ and *M*_n,abs_ (Fig. 5C and D). As expected, the polymerization rate decreased at larger scales (*k*^app^_p_ = 4.46 × 10^−2^ min^−1^ for 10 mL *vs.* 0.83 × 10^−2^ min^−1^ for 250 mL, respectively) (Fig. S10†). This is attributed to the reduced light penetration and greater oxygen diffusion caused by the larger flask size. Notably, comparable dispersity (*Đ*) values were observed in polymerizations performed at all scales, indicating that upscaling does not compromise control over the polymerization process. The ability to polymerize in the presence of oxygen and radical inhibitors is crucial for expanding the applicability of RAFT polymerization to industrial settings. Large-scale deoxygenation and inhibitor removal processes are often cumbersome and expensive, making our method a more practical and cost-effective alternative.

### Expanding the monomer scope of photo-RAFT polymerization

**Fig. 6:**
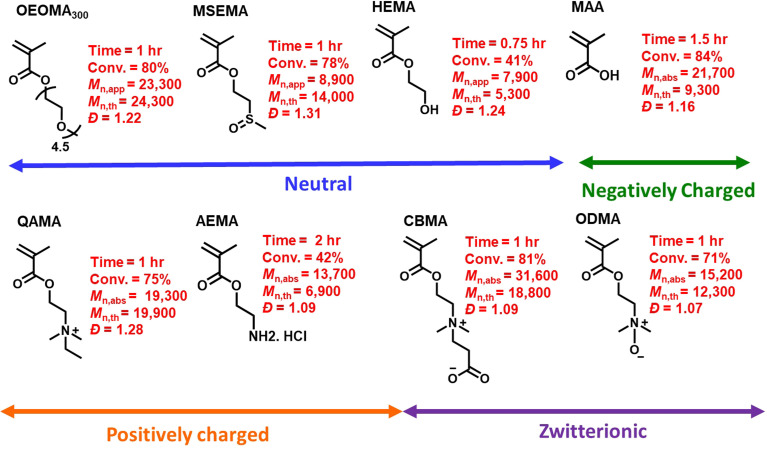
The monomer scope of photo-RAFT polymerization. Conditions for all polymerizations: [monomer]_0_/[CPADB]_0_/[SP]_0_ = 100/1/85 in water/DMSO (9/1 v/v) mixture, [monomer]_0_ = 300 mM. Apparent molecular weight (*M*_n,app_) and dispersity (*Đ*) were determined by SEC analysis (DMF as eluent) calibrated with poly(methyl methacrylate) standards. Absolute molecular weight (*M*_n,abs_) was determined by SEC analysis using d*n*/d*c* values of PMAA = 0.135, PQAMA = 0.145, PAEMA = 0.164, PCBMA = 0.155 and PODMA = 0.158.

Under the optimized conditions, the monomer scope of photo-RAFT polymerization was expanded by testing a range of hydrophilic methacrylate monomers (Fig. 6 and S11–S13†). Using [monomer]_0_/[CPADB]_0_ at a molar ratio of 100/1, photo-RAFT polymerizations were successfully achieved for a variety of monomers, including neutral (*i.e.*, oligo(ethylene oxide) methyl ether methacrylate (average *M*_n_ = 300, OEOMA_300_, 4.5 average number of repeating units), 2-(methylsulfinyl)ethyl methacrylate (MSEMA), and 2-hydroxyethyl methacrylate (HEMA)), negatively charged (*i.e.*, methacrylic acid (MAA)), positively charged (*i.e.*, 2-aminoethyl methacrylate hydrochloride (AEMA) and quaternized diethylaminoethyl methacrylate (QAMA)) as well as a zwitterionic monomers (*i.e.*, 3-[[2(methacryloyloxy)ethyl] dimethylammonio]propionate (CBMA) and *N*-oxide-*N*,*N*-dimethylaminoethyl methacrylate (ODMA)). The polymerizations of all monomers resulted in high conversions (>40%), and polymers with relatively narrow molecular weight distributions (*Đ* ≤ 1.31) and monomodal SEC traces. Remarkably, AEMA, which is a challenging monomer for conventional RAFT polymerization,^91^ was readily polymerized by this technique. This could be attributed to the acidic pH (3.9) of the polymerization mixture, which led to protonation of amines. Oxygen tolerant RAFT polymerization of MSEMA, which is reactive toward the reactive oxygen species generated in PET-RAFT, was also successful.^77,92^ Additionally, a promising and emerging zwitterionic monomer, ODMA,^93,94^ was successfully polymerized by photo-RDRDP for the first time. The extension of SP-RAFT polymerization to a hydrophilic acrylate (*i.e.*, oligo(ethylene oxide) methyl ether acrylate, OEOA_480_) with a trithiocarbonate RAFT agent (4-cyano-4-(((ethylthio)carbonothioyl)thio)pentanoic acid) was also successfully demonstrated (Table S4†).

### Protein–polymer hybrid synthesis and enzymatic activity analysis

**Fig. 7:**
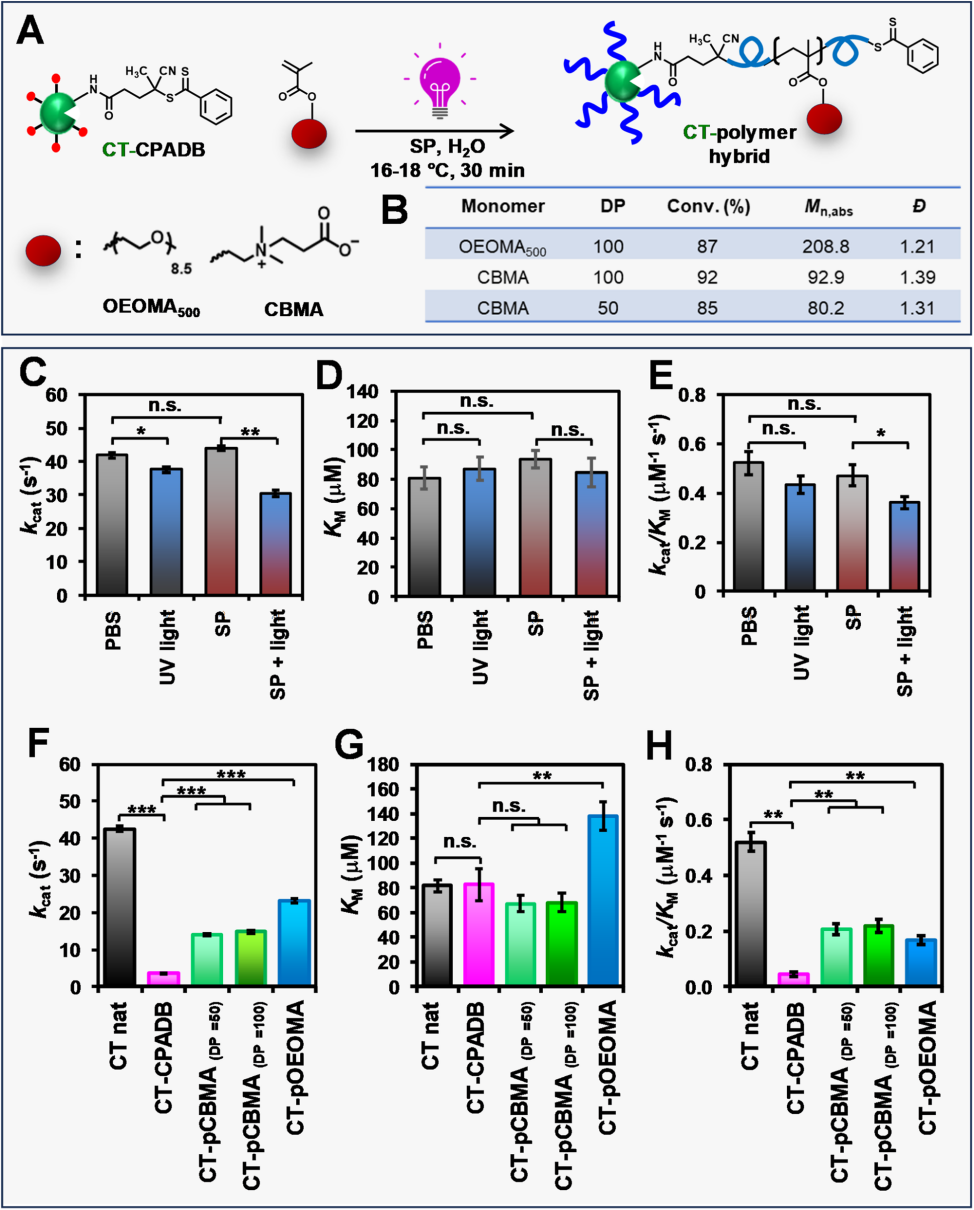
Preparation of CT-PCBMA and CT-POEOMA_500_ hybrids by photo-RAFT with SP and UV light (A). Table of properties of synthesized PPHs (B). Michaelis–Menten parameters of CT after treatment with PBS (control), light, SP, and SP with light (C and E), and CT, CT-CPADB and CT-polymer hybrids (F–H) on substrate suc-AAPF-pNA: *k*_cat_ values (C and F), *K*_M_ values (D and G), and enzymatic efficiency (*k*_cat_/*K*_M_) values (E and H). Data are presented as the mean ± standard deviation (SD) of three (C–E) and four experiments (F–H). Mean differences between experimental groups were tested with the unpaired *t*-test. Values were significantly different at the *p < 0.05, **p < 0.01, ***p < 0.001, or n.s. = not significant. Conditions for PPH synthesis: [monomer]_0_/[CPADB]_0_ = 50–100/1 in water (without DMSO) with SP (128 mM). The polymerizations were performed in open-to-air vials using a Lumidox photoreactor (395 nm, 165 mW cm^−2^) with cooling (15–18 °C) and stirring (300 rpm) for 0.5 h. Conversions were determined by ^1^H-NMR. Absolute molecular weight (*M*_n,abs_) and dispersity (*Đ*) were determined using SEC coupled with a MALS detector.

The applicability of RAFT polymerization with SP for PPH synthesis was demonstrated by “grafting from” chymotrypsin (CT) as a model protein. PI-RAFT polymerization with blue and green irradiation was not applied to PPH synthesis as it was only effective at high monomer concentrations (≥800 mM), which could be detrimental to proteins.^95^ Prior to conducting photo-RAFT polymerization, the effect of sodium pyruvate (SP) and UV irradiation on the activity of native CT was evaluated by measuring Michaelis–Menten parameters. Native CT was irradiated with light (395 nm, 165 mW cm^−2^) in the presence of SP (128 mM), and the enzymatic activity towards a *N*-Suc-Ala-Ala-Pro-Phe-pNA peptide substrate was measured by calorimetric assay and compared with that of CT dissolved in phosphate-buffered saline (PBS) and exposed to UV irradiation. The enzymatic activity of CT incubated in SP was comparable to that of the control CT in PBS in the absence of light (Fig. 7C–E and Table S5†), indicating that the relatively high concentrations of SP did not affect the activity of CT. However, the exposure of native CT to UV irradiation, either in PBS or SP solution, resulted in a 15% and 25% decrease in enzymatic activity, respectively. UV irradiation in the presence of SP did not affect the Michaelis constant (*K*_M_) of native CT, but induced a partial loss of enzyme activity, due to its effect on the turnover (*k*_cat_). We expected that the loss of enzyme efficiency due to UV irradiation in proteins grafted by polymers would not be so significant, so we continued to synthesize other PPHs.

To obtain PPHs, CT was functionalized with CPADB in a two-step procedure. First, *N*-hydroxy succinimide (NHS)-activated CPADB was synthesized (Scheme S2 and Fig. S14†). The activated CPADB was then conjugated to the lysine residues on CT, resulting in a CT-CPADB macro-RAFT agent with 11.1 lysine residues functionalized with CPADB (Scheme S3†). Using CT-CPADB as a macroinitiator, polymerizations of two hydrophilic monomers, OEOMA_500_ and CBMA, were conducted (Fig. 7A). To minimize the detrimental effects of elevated temperatures on proteins, all the polymerizations were carried out at 15–18 °C in a Lumidox photoreactor (395 nm, 165 mW cm^−2^) using open-cap vials as the reaction vessels. In addition, polymerization reactions were stirred to ensure the homogeneity of solutions. The total organic content remained ≤16% as no DMSO was used. After 30 min of irradiation, the polymerizations were stopped, and the bioconjugates were characterized by ^1^H-NMR and SEC coupled with a multi-angle light scattering (MALS) detector (Fig. 7B). When OEOMA_500_ was used as the monomer, the polymerization achieved a conversion of >85%. The polymerization remained fully oxygen-tolerant and exhibited a monomodal trace (Fig. S15†) with a higher absolute molecular weight (*M*_n,abs_ = 208 800) compared to that of CT-CPADB (*M*_n,abs_ = 28 400). Furthermore, our method was successfully extended to the zwitterionic CBMA monomer, which has reduced impact on enzymatic activity.^96^ Polymerization of CBMA from CT-CPADB at target DPs of 50 and 100 reached high monomer conversions (85% and 92%, respectively), maintained moderately low dispersities (1.39 and 1.31, respectively), and exhibited monomodal SEC traces (Fig. S15†).

Trithiocarbonates are typically used to functionalize proteins for RAFT polymerization due to their superior hydrolytic stability.^8^ We were interested in expanding PPH synthesis to dithioesters, which offer comparable control over the polymerization of methacrylate monomers. This necessitated a careful investigation of the dithioester conjugation effect on the enzymatic activity of CT. After functionalization of CT with CPADB, the enzymatic efficiency (*k*_cat_/*K*_M_) decreased by approximately 12-fold compared to that of native CT (Fig. 7F–H and Table S6†). CPADB on the CT surface had a negligible effect on substrate affinity (Fig. 7G) but had a strong effect on *k*_cat_ (Fig. 7F). This is attributed to changes in the charge and hydrophobicity of the CT surface by hydrophobic CPADB. Such changes resulted in distorting the CT active site and significantly reduced *k*_cat_. Interestingly, the polymerization of OEOMA_500_ and CBMA increased the enzymatic activity by 4–5 fold compared to that of CT-CPADB. This can be explained by the fact that the grafted polymer detaches the hydrophobic CPADB from the CT surface, recovering the CT active site and increasing the substrate turnover (Fig. 7F). Hybrids modified with a hydrophilic zwitterionic polymer (PCBMA) showed no change in substrate affinity, while hybrids modified with POEOMA_500_, which has larger side chains, had higher *K*_M_ values. This is because the oligo(ethylene oxide) group, which is more hydrophobic than zwitterionic carboxybetaine and has a large side chain, inhibits access of the highly hydrophobic peptide substrates to the active site. Overall, photo-RAFT polymerization using SP did not engender a large decrease in *k*_cat_, and it maintained a catalytic efficiency equivalent to that of CT-PCBMA and CT-POEOMA_500_ hybrids previously prepared by ATRP.^96,97^ In addition, the enzyme activity lost by modification of RAFT agent was restored by modification with a biocompatible polymer such as PCBMA.

## Conclusions

A photo-RAFT polymerization technique was developed using water-soluble and biocompatible PADs, such as sodium pyruvate (SP), as photoinitiators or promoters. This method enabled rapid and fully oxygen-tolerant polymerization in aqueous media, overcoming the limitations of some conventional aqueous RAFT polymerization techniques. Under UV irradiation (370 nm), SP decomposes to form CO_2_ and radicals, initiating polymerization. Under blue (450 nm) or green (525 nm) irradiation, SP enhanced the polymerization rate and decreased the induction period by interaction with excited state CPADB. Photo-RAFT polymerization using SP controlled the polymerization of various hydrophilic methacrylates and upscaled polymerization, with excellent chain end fidelity and temporal control. Furthermore, this method was extended to the synthesis of PPHs of POEOMA_500_ and PCBMA. The results revealed a significant improvement in the catalytic efficiency of the PPH compared to proteins functionalized with CPADB.

## Data availability

Additional data and detailed experimental details are available in the ESI.†

## Author contributions

AJ, MC, RB, KK, XH, FD designed experiments and collected experimental data. HM carried out protein's activity assays. AL and GS performed mechanistic experiments and proposed mechanisms. KM supervised the research. The manuscript was written through contributions of all authors.

## Conflicts of interest

The authors declare no competing financial interest.

## Supplementary Material

SC-015-D4SC01409J-s001
