# Supplementary material for: Aqueous photo-RAFT polymerization under ambient conditions: synthesis of protein–polymer hybrids in open air
Source: Chem Sci. 2024 May 24;15(25):9742–55. doi: 10.1039/d4sc01409j (PMC11206215; doi:10.1039/d4sc01409j)
Supplement: SC-015-D4SC01409J-s001 [file SC-015-D4SC01409J-s001.pdf]

Electronic Supplementary Material (ESI) for Chemical Science.
This journal is © The Royal Society of Chemistry 2024

Aqueous Photo-RAFT Polymerization under Ambient Conditions: Synthesis of Protein-Polymer Hybrids in Open Air

*Arman Moini Jazani,[a] Hironobu Murata,[a] Martin Cvek,[a,b] Anna Lewandowska-Andralojc,[c,d] Roksana Bernat,[a,e] Kriti Kapil,[a] Xiaolei Hu,[a] Ferdinando De Luca Bossa,[a] Grzegorz Szczepaniak,[a,f] and Krzysztof Matyjaszewski[a]**

[a]*Department of Chemistry, Carnegie Mellon University, 4400 Fifth Avenue, Pittsburgh, PA 15213, United States.*

[b]*Centre of Polymer Systems, Tomas Bata University in Zlin, Trida T. Bati 5678, 76001 Zlin, Czech Republic.*

[c]*Faculty of Chemistry, Adam Mickiewicz University, Uniwersytetu Poznanskiego 8, 61-614 Poznan, Poland.*

[d]*Center for Advanced Technology, Adam Mickiewicz University, Uniwersytetu Poznanskiego 10, 61-614 Poznan, Poland.*

[e]*Institute of Materials Engineering, University of Silesia, 75 Pulku Piechoty 1A, 41-500 Chorzow, Poland.*

[f]*Faculty of Chemistry, University of Warsaw, Pasteura 1, 02-093 Warsaw, Poland.*

Table of contents

Experimental Details 2

Materials 2

Instrumentation 3

Table of literature examples of oxygen-tolerant RAFT polymerization in water 6

Procedures for photo-RAFT polymerizations of $OEOMA_{500}$ 8

Genral procedure for photo-RAFT polymerization with SP 8

Genral procedure for photo-RAFT polymerization without SP 8

General procedure for photo-RAFT polymerization with SP and without DMSO 8

Kinetics of photo-RAFT polymerization under UV, blue and green light 9

SP photodecompisiton analysis by ^{13}C-NMR and ^{1}H-NMR 11

Discusion on the role of SP in visible light induced PI-RAFT polymerization 12

Temporal control of photo-RAFT polymerization……………………………………………16

In situ chain extension experiment………………………………………………………....16

Procedure for photo-RAFT polymerization with different Target DPs…………………..……17

Scale up of photo-RAFT polymerization…………………………………………………19

Expanding monomer scope of photo-RAFT polymerization..……………………………………21

Synthesis of ODMA...……………...……………………………………………………21

Synthesis of MSEMA…………………………………………………………………….22

Polymerization of $OEOA_{480}$……………………………………………………..……23

Protein-polymer hybrids synthesis and characterization 24

Impact of UV light irradiation with/without SP on enzymatic activity of CT………………..24

Synthesis of CPADB-NHS...24

Synthesis of CT-CPADB macro-RAFT agent 25

Synthesis of protein-polymer hybrids 26

PPH kinetic assay 27

References...28

Experimental Details

Materials

All chemicals were received from commercial vendors and used as received unless otherwise noted: 4-cyano-4-(phenylcarbonothioylthio)pentanoic acid (CPADB, 97%) from Strem Chemicals; 4-cyano-4-(((ethylthio)carbonothioyl)thio)pentanoic acid (95%) from Ambeed; sodium pyruvate (SP, >97%), ethyl pyruvate (EP, >97%), pyruvic acid (PA, >97%), 3-[[2(methacryloyloxy)ethyl]dimethylammonio]propionate (CBMA, >98.0%) from Tokyo Chemical Industry (TCI); oxalic acid (OA, 98%) from thermos scientific; sodium oxalate (SO, 95%) from Combi-blocks; water (HPLC grade), dimethyl sulfoxide (DMSO, ≥ 99.7%), hydrogen

peroxide (H_2O_2, 30%), and magnesium sulfate anhydrous ($MgSO_4$) from Fisher Scientific; 1-ethyl-3-(3′-dimethylaminopropyl)carbodiimide hydrochloride (EDC, 99%) from Oakwood Chemical; oligo(ethylene oxide) methyl ether methacrylate (average M_n = 500, 8.5 average number of repeating units, $OEOMA_{500}$ and average M_n = 300, 4.5 average number of repeating units), $OEOMA_{300}$), oligo(ethylene oxide) methyl ether acrylate (average M_n = 480, 8.5 average number of repeating units, $OEOA_{480}$), 2-hydroxyethyl methacrylate (HEMA, ≥ 99%), 2-aminoethyl methacrylate hydrochloride (AEMA.HCl, 90%), methacrylic acid (MAA, 99%), 2-(dimethylamino)ethyl methacrylate (DMAEMA, 98%), N-hydroxysuccinimide (NHS, 98%), 4-(dimethylamino)pyridine (DMAP, ≥ 99%), 2-(methylthio)ethanol (99%), N,N′-diisopropylcarbodiimide (DIC, 99%), and α-chymotrypsin (CT, bovine pancreas) from Sigma Aldrich; N-succinyl-L-Ala-L-Ala-L-Pro-L-Phe-p-nitroanilide (sucAAPFpNA) from Bachem.

$OEOMA_{500}$, $OEOMA_{300}$ and $OEOA_{480}$ were passed through a column of basic alumina to remove inhibitor prior to use. Other monomers were used without inhibitor removal.

Instrumentation

^{1}H-NMR spectra were recorded using a Bruker Avance III 500 MHz spectrometer. D_2O was used as the NMR solvent.

^{13}C-NMR spectra were recorded Bruker Avance NEO 500 MHz NMR Instrument. D_2O was used as the NMR solvent.

Molecular weight (M_n) and molecular weight distribution ($Đ$) of $POEOMA_{500}$, $POEOMA_{300}$, $POEOA_{480}$, PMSEMA, and PHEMA were determined by gel permeation chromatography (GPC) with DMF as an eluent. An Agilent GPC was equipped with an RI detector and PSS columns (Styrogel 10^5, 10^3, 10^2 Å) and run at 50 °C at the flow rate of 1 mL/min. Apparent molecular weight ($M_{n,\,app}$) was obtained by using linear poly(methyl methacrylate) calibration standard. The clear solutions were filtered using a 0.40 mm PTFE filter to remove any DMF-insoluble species. Absolute molecular weight ($M_{n,\,abs}$) was determined using dn/dc values reported in Table S1.

SEC-MALS measurements of PMAA, PQAMA, PAEMA, PCBMA and PODMA were performed using Agilent SEC system (Agilent, 1260 Infinity II) coupled with MALS, DLS, UV, Viscometer and RI detectors (Wyatt Technology, USA). Measurements were performed using Waters Ultra hydrogel Linear column with PBS (pH = 7.4) for PCBMA and PODMA, 10 mM

sodium phosphate and 100 mM NaCl (pH = 8.0) for PMAA, and 100 mM sodium phosphate with 0.2 vol% of trifluoroacetic acid (pH = 2) for PAEMA and PQAMA as the eluent at room temperature and the flow rate of 0.5 mL/min. Absolute molecular weight ($M_{n, abs}$) was determined using dn/dc values reported in Table S1.

UV-Vis spectra were recorded using an Agilent 8453 spectrophotometer.

Femtosecond transient absorption measurements were conducted using the Solstice Ti:sapphire regenerative amplifier from Spectra Physics and an optical detection system provided by Ultrafast Systems (Helios). The source for the pump and probe pulses was the fundamental emission at 800 nm. The fundamental output was split into two beams: a pump (95%) and a probe (5%). The pump beam was directed through the TOPAS-Prime automated optical parametric amplifier from the Spectra Physics to obtain the desired excitation wavelength in the range 290-2600 nm. The probe beam was directed to the Helios: a CCD-based pump-probe TA spectrometer from Ultrafast Systems LLC with an optical delay line allowing delay between the pump and probe up to 3.2 ns. For the detection of the transients, a white light continuum was used, which was generated from 5% of the fundamental beam by passing it through a sapphire or calcium fluoride crystal. For transient UV-vis measurements a quartz cell with 2 mm optical path of solution was used with the absorbance of about 0.2 at the excitation wavelength, the sample solution was stirred by a Teflon-coated bar. All experiments were performed at room temperature. Global analysis of the transient absorption data was made using the Surface Explorer software (Ultrafast Systems).
Oxygen measurements were performed using FireStingGO2 pocket oxygen meter and a solvent-resistant oxygen probe purchased from PyroScience. The probe was calibrated with solution of Oxcal 0% O_2 calibration capsules in water and normal water. The probe was inserted into an open cap vial while being irradiated by light. The data was calibrated by dividing each value by the maximum values of oxygen concentrations.

Light intensity measurements were done by using Thorlabs PM100D digital handheld optical power and energy meter.

In all the cases, RAFT polymerizations were performed in the open cap vials placed in the photo-reactor (PhotoRedoxBox, HepatoChem, USA). UV (370 nm, 6.5 mW/cm^2), blue (450 nm, 21.5 mW/cm^2), and green light (525 nm, 20.0 mW/cm^2) light sources were purchased from

Kessil with adjustable intensity of irradiation. The light intensity values reported on the light source did not correspond to the measured values.

Table S1. Measured dn/dc values of polymers used for determining absolute molecular weight ($M_{n,\ abs}$).

Polymers	dn/dc
$POEOMA_{500}$	0.055
PMAA	0.135
PQAMA	0.145
PAEMA	0.164
PCBMA	0.155
PODMA	0.158

Table of literature examples of oxygen-tolerant RAFT polymerization in water

Table S2. Literature examples of oxygen tolerant RAFT polymerization in water.

RAFT	*Corresponding Author*	*Oxygen removal Mechanism*	*Conditions*	*References*
1	Matyjaszewski *et al.*	Photoradical initiator and Photoiniferter	Open vessel, with stirring	This work
2	Stevens *et al.*	Enzyme degassing	Open vessel	[1]
3	Stevens *et al.*	Enzyme degassing	Open vessel	[2]
4	An *et al.*	Enzyme degassing	Open vessel, with stirring	[3]
5	Zhang *et al.*	Enzyme degassing	Conditions were not described	[4]
6	An *et al.*	Enzyme degassing	Conditions were not described	[5]
7	Rowan *et al.*	Enzyme degassing	Open vessel, with stirring	[6]
8	Qiao *et al.*	Enzyme degassing	Sealed vessel, with stirring	[7]
9	An *et al.*	Enzyme degassing	Sealed vessel	[8]
10	Gurnani *et al.*	Enzyme degassing	Open vessel, with stirring	[9]
11	Weil *et al.*	Enzyme degassing	Sealed vessel	[10]
12	Perrier *et al.*	Thermal radical initiator	Open vessel	[11]
13	Tao *et al.*	Thermal radical	Open vessel	[12]

		initiator		
14	Cooper-White *et al.*	Thermal radical initiator	Open vessel	[13]
15	Boyer *et al.*	PET-RAFT	Open vessel	[14]
16	Zhu *et al.*	PET-RAFT	Open vessel	[15]
17	Perez-Mercader *et al.*	PET-RAFT	Sealed vessel	[16]
18	Boyer *et al.*	PET-RAFT	Sealed vessel	[17]
19	Zou *et al.*	PET-RAFT	Open vessel	[18]
20	Boyer *et al.*	PET-RAFT	Sealed vessel	[19]
21	Kwon *et al.*	PET-RAFT	Sealed vessel	[20]
22	Geng *et al.*	PET-RAFT	Sealed vessel	[21]
23	Cai *et al.*	PET-RAFT	Sealed vessel	[22]
24	Hou *et al.*	PET-RAFT	Sealed vessel	[23]
25	Kwon *et al.*	PET-RAFT	Sealed vessel	[24]
26	Deng *et al.*	PET-RAFT	Sealed vessel	[25]
27	Hou *et al.*	PET-RAFT	Open vessel, with stirring	[26]
28	Guo *et al.*	PET-RAFT	Open vessel	[27]
29	Matyjaszewski *et al.*	PET-RAFT	Sealed vessel	[28]

Procedures for photo-RAFT polymerization of $OEOMA_{500}$

General procedure for photo-RAFT polymerization with SP

For polymerization at $[OEOMA_{500}]_0$ = 300 mM, $OEOMA_{500}$ (0.75 g, 1.5 mmol) and SP (0.14 g, 1.3 mmol) were mixed in a volumetric flask (5 mL), followed by the addition of CPADB (4.2 mg, 0.015 mmol) from a stock solution. For polymerization at $[OEOMA_{500}]_0$ = 800 mM, $OEOMA_{500}$ (2.0 g, 4.0 mmol) and SP (0.37 g, 3.4 mmol) were mixed in a volumetric flask (5 mL), followed by the addition of CPADB (11.2 mg, 0.04 mmol) from the stock solution.

After adjusting the DMSO volume to (500 µL, 10% *v/v*), the volumetric flask was topped up with water. The mixture was vortexed for 3 min, transferred to an open-to-air glass vial (1 mL), and irradiated with different lights to initiate polymerization. Polymerizations were stopped by turning the light off and residues were mixed in characterization solvents (DMF for GPC and D_2O for ^{1}H-NMR).

Similar procedures were performed for carrying out polymerization of other pyruvic acid derivatives (PADs) except for the use of EP (0.15 g, 1.3 mmol), PA (0.11 g, 1.3 mmol), OA (0.11 g, 1.3 mmol) and SO (0.085 g, 0.63 mmol).

General procedure for photo-RAFT polymerization without SP

For polymerization at $[OEOMA_{500}]_0$ = 300 mM, $OEOMA_{500}$ (750 mg, 1.5 mmol) was mixed with CPADB (4.2 mg, 0.015 mmol) from a stock solution into a volumetric flask (5 mL), and for polymerization at $[OEOMA_{500}]_0$ = 800 mM, $OEOMA_{500}$ (2.0 g, 4 mmol) was mixed with CPADB (11.2 mg, 0.04 mmol) from the stock solution in a volumetric flask (5 mL).

After adjusting the DMSO volume (500 µL, 10% v/v), the volumetric flask (5 mL) was topped up with water. The mixture was vortexed for 3 minutes, transferred to a 1 mL open-cap glass vial, and was irradiated with different lights (UV, green and blue) to initiate the polymerization. Polymerization was stopped by turning the light off and dilution of samples in characterization solvent (DMF for GPC or D_2O for NMR).

General procedure for photo-RAFT polymerization with SP and without DMSO

$OEOMA_{500}$ (750 mg, 1.5 mmol) and SP were mixed in a volumetric flask (5.0 mL), followed by the addition of CPADB (4.2 mg, 0.015 mol). The mixture was heated up to 40 °C for 10 min to increase the solubility of CPADB in the mixture. Next, the volumetric flask (5 mL) was topped up with water. The mixture was vortexed for 10 min, transferred to an open-cap glass vial (1 mL), and irradiated with light to initiate polymerization.

Kinetic analysis of photo-RAFT polymerization

The kinetics of the RAFT polymerizations were monitored under irradiation with three different light sources (UV, blue and green). In the typical reaction under UV light, the mixture was prepared following the aforementioned protocol, and the mixture without SP served as a reference. For the reactions under blue and green lights, the reactant ratios were adjusted as follows: $OEOMA_{500}$ (2.0 g, 800 mM), SP (0.14 g, 255 mM) and CPADB (8.5 mg, 4.0 mM) dissolved in water. The total reaction volume, and the ratio of water and DMSO (9/1 *v/v*) were kept identical. When working under UV light, the aliquots (100 μL) were withdrawn in 10-minute intervals, while a withdrawal period of 60 minutes was selected when carrying out the RAFT polymerizations under blue and green lights. The purified products were analyzed by ^{1}H NMR and GPC techniques.

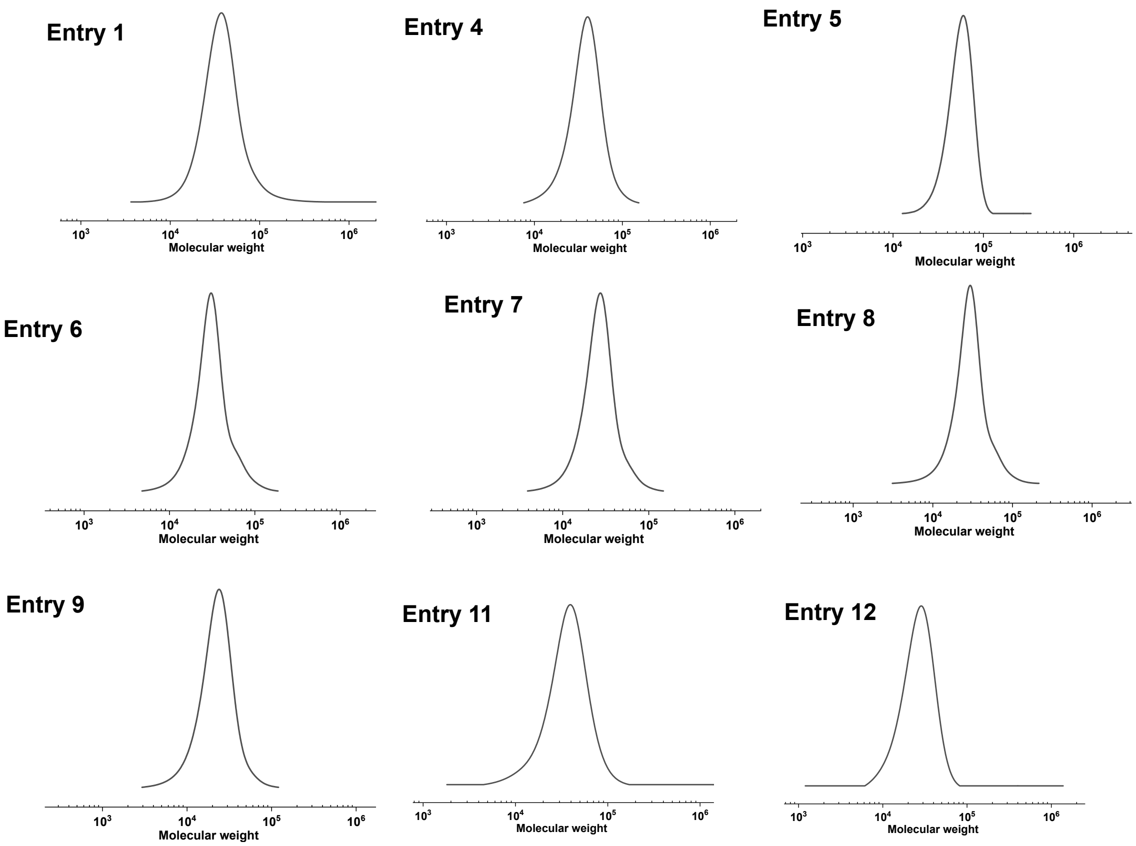

Figure S1. SEC traces of $POEOMA_{500}$ presented in Table 1.

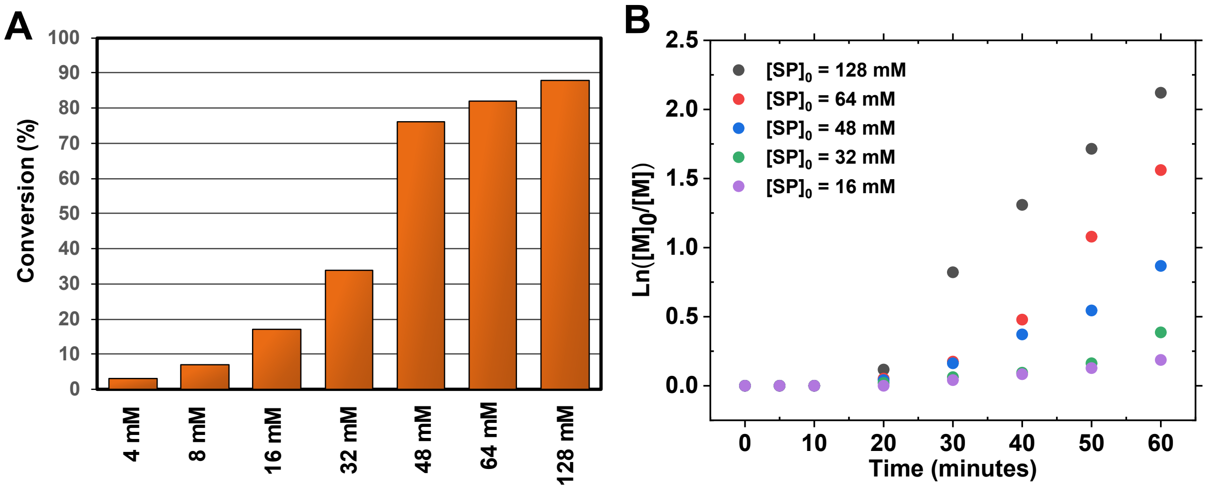

Figure S2. Effect of SP concentration in photo-RAFT polymerization of $OEOMA_{500}$ with SP upon irradiation with UV for 1 h: final conversions (A) and kinetics of polymerization at different SP concentrations (B).

SP photodecomposition analysis by ^{13}C-NMR and ^{1}H-NMR

SP (50 mg, 0.45 mmol) was dissolved in D_2O (1.0 mL) and transferred to an NMR tube. After 4 h UV light irradiation (370 nm, 26.3 mW/cm^2) at room temperature, ^{13}C-NMR and ^{1}H-NMR of the solution was measured and compared to the initial time. The degradation products show major peaks at 1.35 ppm and 2.25 ppm and smaller peaks at 4.05 ppm in ^{1}H-NMR. The degradation products include but not limited to acetic acid, lactic acid, acetoin and 2,3-dimethyl tartaric acid.[29]

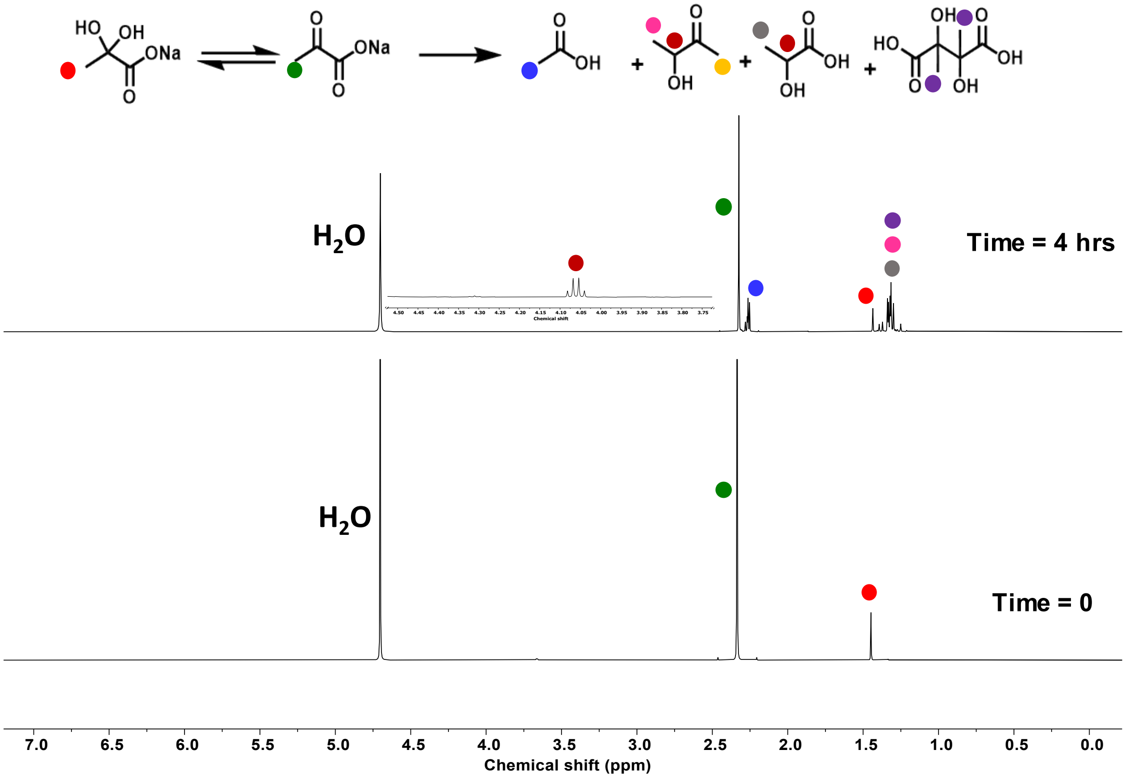

Figure S3. ^{1}H-NMR of the photodecomposition of SP after UV irradiation.

Discussion on the role of SP in visible light induced PI-RAFT polymerization

The observed increase in rate of polymerization, suppression of induction period and enhanced oxygen tolerance in the presence of SP upon visible light irradiation suggest the interaction of SP with ground or excited states of CPADB. This was investigated by several other experiments. First, the polymerization of $OEOMA_{500}$ with blue light irradiation (450 nm) was carried out using different SP concentrations. As shown in Figure S4, the conversions of $OEOMA_{500}$ are proportional to the concentration of SP used for the polymerization and increased with higher SP concentration.

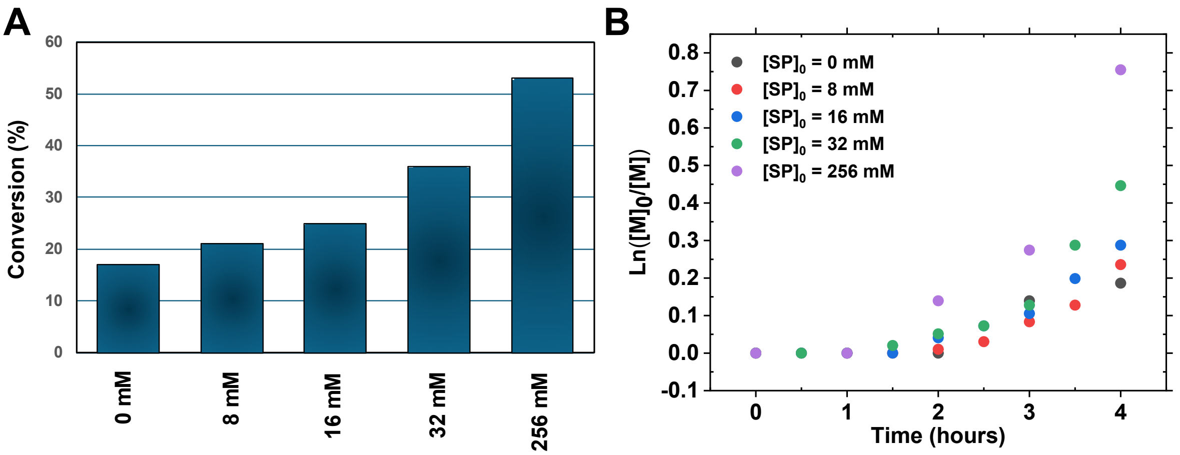

Figure S4. Effect of SP concentration in photo-RAFT polymerization of $OEOMA_{500}$ with SP upon irradiation with blue light for 4 h: final conversions (A) and kinetics of polymerization at different SP concentrations (B).

CPADB exhibited interesting wavelength-dependent photochemistry (Figure S5) and therefore it was of interest to study properties of CPADB excited to S_2 or S_1. Qualitative analysis of the different photochemistry depending on the excitation wavelength was performed by steady-state irradiation experiments using 370 nm and 505 nm LED diode. Steady-state irradiation of CPADB with 370 nm LED diode led to almost complete disappearance of the CPADB absorbance within 3 minutes of irradiation (Figure S5A). Surprisingly, irradiation of the CPADB in the visible range with 505 nm LED diode did not cause any meaningful changes in the UV-VIS spectra even after 4 h of irradiation (Figure S5B). This conclusion is in an agreement with previous work by Thum *et al.* where it was shown that the excitation of dithioesters into their S_1 band (*i.e.,* visible light) results in negligible production of radicals due to the slightly higher bond dissociation energies of C-S bond compared to the lowest singlet and triplet excited state energies of dithioesters.[30]

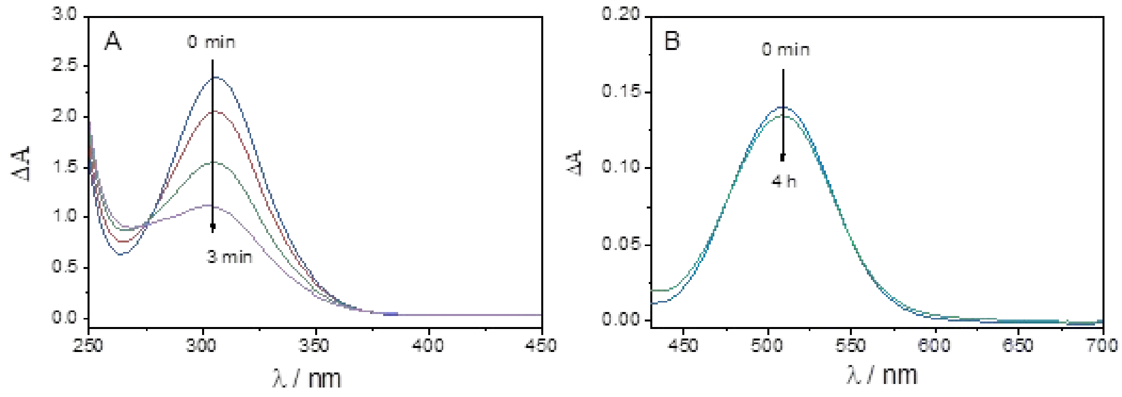

Figure S5. Comparison of decomposition of CPADB in DMSO-water (1:2 *v/v*) under A) 370 nm and B) 505 nm irradiation as measured by UV-Vis spectroscopy.

Unlike SP-free conditions which exhibited nearly identical UV-vis spectra, when the steady state irradiation of CPADB has been performed in the presence of SP (255 mM), the intensity of the CPADB band at 475-575 nm decreases upon light irradiation (Figure S6, red square). This was also accompanied by an increase in overall absorption, which was attributed to the light scattering as some photoproducts of SP and CPADB reaction is possibly not well soluble in this solvent mixture.

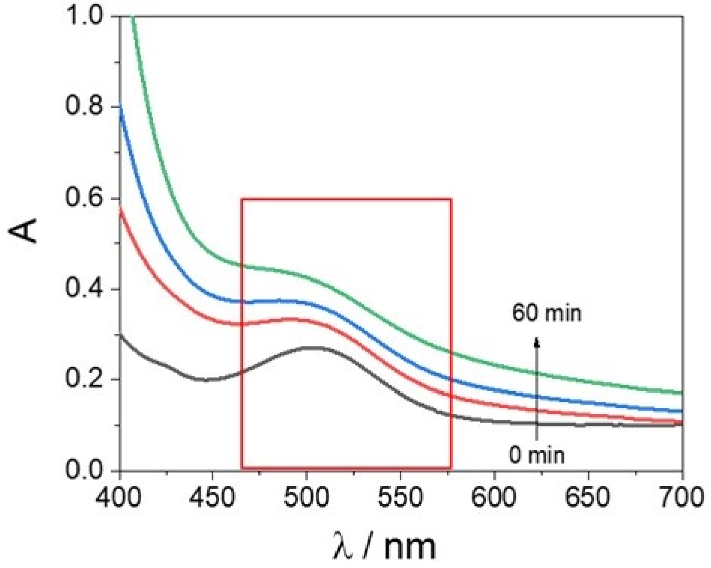

Figure S6. Decomposition of CPADB in DMSO-water (1:2 *v/v*) in the presence of SP (255 mM) under 505 nm irradiation as measured by UV-VIS spectroscopy.

Ultrafast transient absorption experiments were also conducted to monitor the possible influence of the SP on the deactivation pathways of $^{1}S^{*}$ of the CPADB. The transient absorption spectra

recorded for CPADB in the presence of SP just after the excitation is similar to the analogous spectra recorded for CPADB (Figure S7). However as presented in Figure S7B, formation of an additional band in the region of 500-550 nm was detected at longer time delays. Interestingly the new species with the absorption in the 500-550 nm does not decay over the 3 ns period (Figure S8).

By comparing the monitored changes in the UV-vis spectra and ultrafast transient absorption experiments of CPADB with and without SP upon irradiation, it can be concluded that there is an interaction between SP and the photoexcited CPADB into S_1 state. The most possible interaction is the electron transfer from SP to the excited CPADB, followed by decarboxylation of SP and formation of other decomposition products.

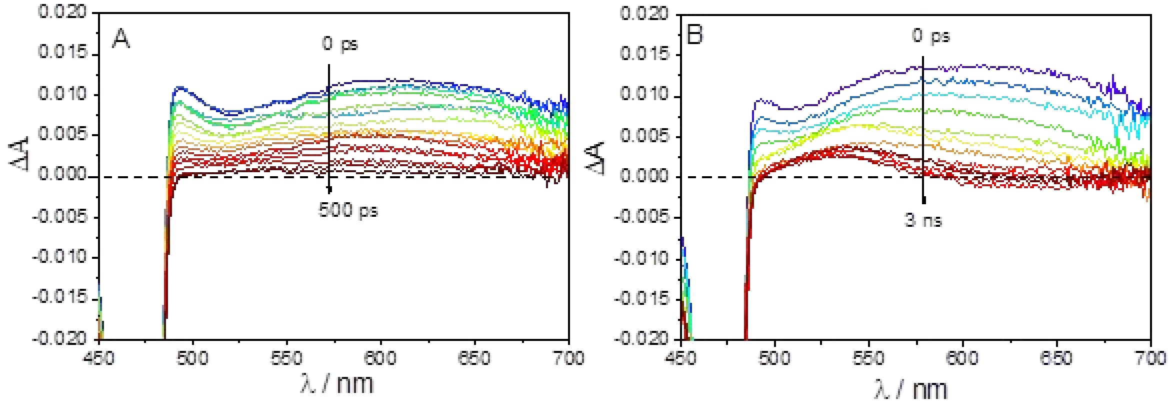

Figure S7. Transient absorption spectra obtained at different time delays for A) CPADB (14 mM) and B) CPADB in the presence of SP (255 mM) in DMSO-water (1:2 *v/v*) following the 470 nm laser excitation.

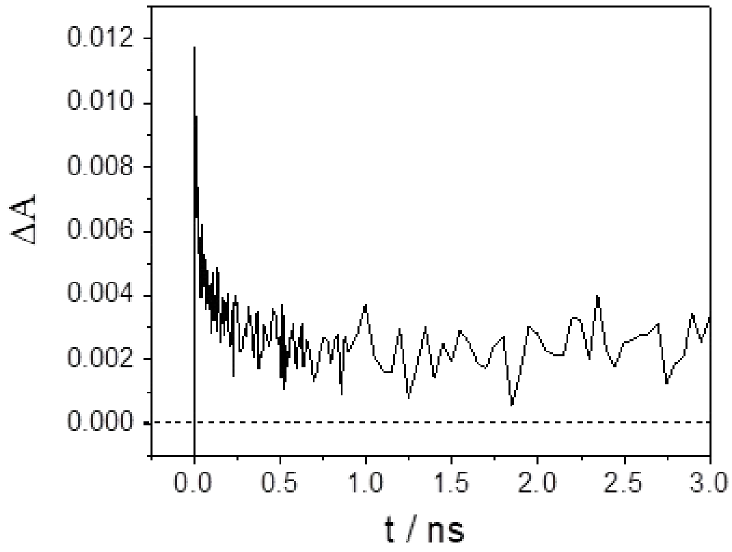

Figure S8. Absorption time profiles at 530 nm measured for the CPADB in the presence of SP (255 mM) following the 470 nm laser excitation.

Temporal control of photo-RAFT polymerization

A vial (4.8 mL) was charged with $OEOMA_{500}$ (543 µL, 300 mM), SP (0.11 g, 255 mM) dissolved in water, and CPADB (3.2 mg, 1.5 mM) in DMSO. The total reaction volume (including monomer) was set to 3.8 mL while adjusting the DMSO content to 10 % *v/v*. The homogeneity was achieved by a high-speed vortex, and the opened vial was placed into an air-conditioned photo-reactor fitted with the UV-light source. The reactor was exposed to periodic irradiation/dark phases that each lasted for around 20 min. Before withdrawing the aliquots (100 µL), the reactor was shortly stirred using a vortex device to ensure homogeneity prior to analyzing by ^{1}H NMR and GPC techniques.

***In situ* chain extension experiment**

The *in-situ* chain extension experiment was carried out by targeting DP = 50 for the first block and DP = 150 for the second block. Briefly, $OEOMA_{500}$ (0.25 g, 0.5 mmol) and SP (28.2 mg, 0.25 mmol) were mixed in a volumetric flask (1.0 mL), followed by the addition of CPADB (2.8 mg, 0.010 mmol) stock solution in DMSO. After adjusting the DMSO volume (100 µL, 10% *v/v*), the volumetric flask (1.0 mL) was topped up with water. The mixture was vortexed for 3 minutes, transferred to a 1 mL open-cap glass vial, and was irradiated with light (3 h) to carry out the polymerization. Polymerization was stopped by turning the light off, and aliquot of polymerization (250 µl) was mixed with a fresh $OEOMA_{500}$ (62.5 mg, 0.12 mmol) and water (250 µL) in a glass vial (1 mL). The mixture was vortexed and irradiated with UV light for 0.5 h.

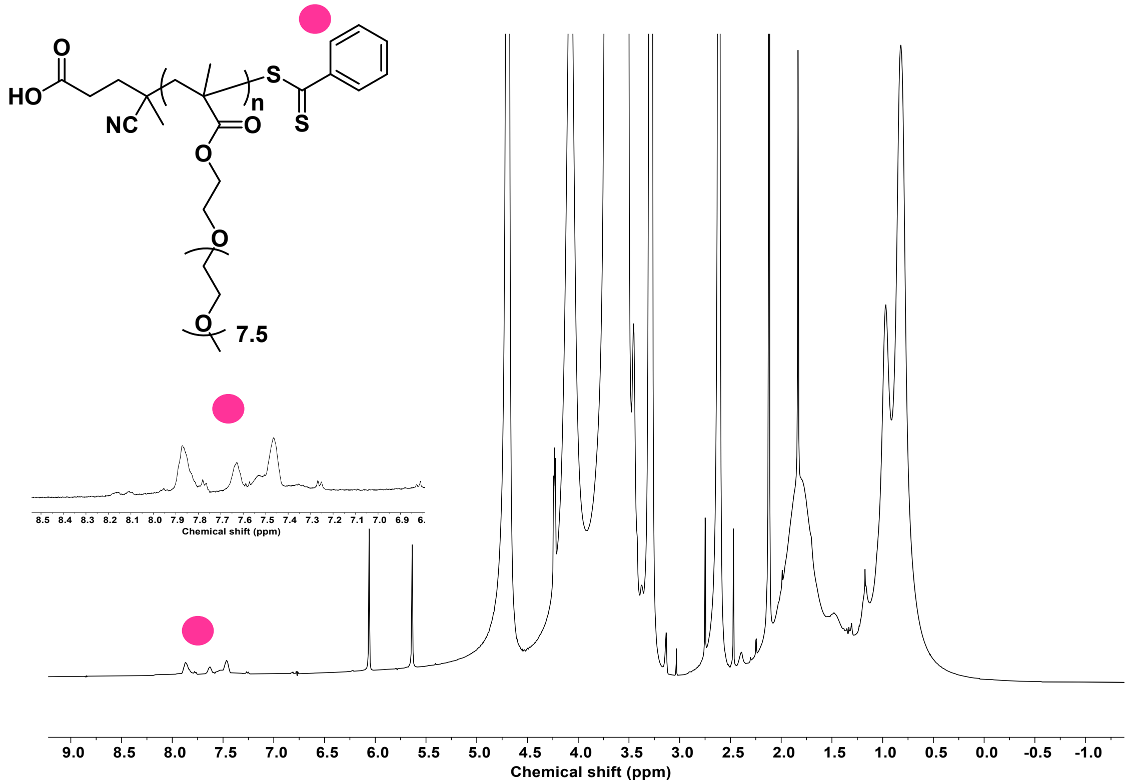

Figure S9. ^{1}H-NMR of purified $POEOMA_{500}$ synthesized by photo-RAFT polymerization with SP.

Procedure for photo-RAFT polymerization at different target DP

The target degrees of polymerization (DP) were varied by adjusting the CPADB concentration, while the concentrations of all the other components, such as $OEOMA_{500}$ (300-800 mM), SP (128-256 mM), and DMSO (10% *v/v*) were kept constant. After preparing the mixture, it was vortexed for 3 min, transferred into five open-to-air glass vials (1 mL), and was irradiated with UV light to initiate polymerization.

For polymerization at $[OEOMA_{500}]_0$ = 300 mM, polymerization mixtures (5 mL) were prepared by mixing $OEOMA_{500}$ (0.75 g, 1.5 mmol) and SP (140.2 mg, 1.27 mmol), followed by the addition of DMSO (500 µL, 10% *v/v*) and water. After dividing the mixture into five different vials (1 mL), CPADB stock solution (12.4 mg/mL) at a different ratio (0.006 mmol, 0.0015 mmol, 0.0008 mmol, 0.0004 mmol) were added to obtain the target DP = 50, 200, 400, and 800, respectively.

For polymerization at $[OEOMA_{500}]_0 = 500$ mM, polymerization mixtures (5 mL) were prepared by mixing $OEOMA_{500}$ (1.25 g, 2.5 mmol) and SP (70.4 mg, 0.64 mmol), followed by the addition of DMSO (500 µL, 10% *v/v*) and water. After dividing the mixture into 5 different vials (1 mL), CPADB stock solution (60 mg/mL) at a different ratio (0.01 mmol, 0.005 mmol, 0.0025 mmol, 0.00125, 0.000625 mmol) were added to obtain the target DP = 50, 100, 200, 400, and 800, respectively.

For polymerization at $[OEOMA_{500}]_0 = 800$ mM, polymerization mixtures (5 mL) were prepared by mixing $OEOMA_{500}$ (2.0 g, 4 mmol) and SP (0.14 g, 1.3 mmol), followed by the addition of DMSO (500 µL, 10% *v/v*) and water. After dividing the mixture into 5 different 1 mL vials, CPADB stock solution (46 mg/mL) at a different ratio (0.016 mmol, 0.004 mmol, 0.001 mmol) were added to obtain the target DP = 50, 200, 400, and 800, respectively.

Table S3. Properties of $POEOMA_{500}$ synthesized with different target DP and concentrations of $OEOMA_{500}$ by photo-RAFT polymerization in the presence of CPADB and SP.[a)]

DP	$[SP]_0$ mM	$[OEOMA_{500}]_0$ mM	Time (h)	^{b}Conv. (%)	$^cM_{n,th}$	$^cM_{n,app}$	$^c Đ$
50	256	300	1	19	-	-	-
200	256	300	1	>95	95,000	100,900	1.36
400	256	300	1	>95	190,000	145,000	1.65
800	256	300	1	>95	380,000	134,400	2.45
50	128	500	3	55	14,000	13,850	1.24
100	128	500	1.3	70	35,300	24,500	1.23
200	128	500	1	90	90,300	48,700	1.27
400	128	500	1	>95	190,300	110,000	1.60
800	128	500	0.5	>95	380,300	156,200	1.87
50	256	800	2	75	18,750	22,920	1.23
200	256	800	0.5	85	85,000	75,060	1.44
400	256	800	0.5	90	180,00	211,200	2.09
800	256	800	0.5	gel	-	-	-

[a)] Reactions conditions: $[OEOMA_{500}]_0/[CPADB]_0$= 100/1 in water/DMSO (9/1 *v*/*v*), irradiated with Kessil LEDs (370 nm, 6.5 mW/cm^2) in an open-cap vial. [b)] Monomer conversion was determined by using ^{1}H-NMR spectroscopy. [c)] Molecular weight ($M_{n,app}$) and dispersity ($Đ$) were determined by SEC analysis (DMF as eluent) calibrated with poly(methyl methacrylate) standards.

Scale up of photo-RAFT polymerization

Polymerization at five different scales was carried out in completely open to air round bottom flasks (RBF) of different sizes (10 mL, 25 mL, 50 mL, 100 mL, and 200 mL) and stirred at 250 RPM for 1 h. The mixture was irradiated with UV light. In all cases, monomers without standard inhibitor purification procedures were used for polymerization.

For polymerization at 10 mL, $OEOMA_{500}$ (1.5 g, 3.0 mmol) and SP (0.28 g, 2.5 mmol) were mixed in a 10 mL RBF, followed by the addition of CPADB (8.4 mg, 0.03 mmol). After adding DMSO (1 mL, 10% *v/v*), the RBF was topped up with water.

For polymerization at 25 mL, $OEOMA_{500}$ (3.7 g, 7.5 mmol) and SP (0.70 g, 6.4 mmol) were mixed in a 25 mL RBF, followed by the addition of CPADB (21.0 mg, 0.075 mmol). After adding DMSO (2.5 mL, 10% *v/v*), the RBF was topped up with water.

For polymerization at 50 mL, $OEOMA_{500}$ (7.5 g, 15 mmol) and SP (1.4 g, 13 mmol) were mixed in a 50 mL RBF, followed by the addition of CPADB (42.0 mg, 0.15 mmol). After adding DMSO (5 mL, 10% *v/v*), the RBF was topped up with water.

For polymerization at 100 mL, $OEOMA_{500}$ (15 g, 30 mmol) and SP (2.8 g, 25 mmol) were mixed in a 100 mL RBF, followed by the addition of CPADB (84 mg, 0.3 mmol). After adding DMSO (10 mL, 10% *v/v*), the RBF was topped up with water.

For polymerization at 200 mL, $OEOMA_{500}$ (37 g, 75 mmol) and SP (7.0 g, 64 mmol) were mixed in a 200 mL RBF, followed by the addition of CPADB (0.21 g, 0.75 mmol). After adding DMSO (20 mL, 10% *v/v*), the RBF was topped up with water.

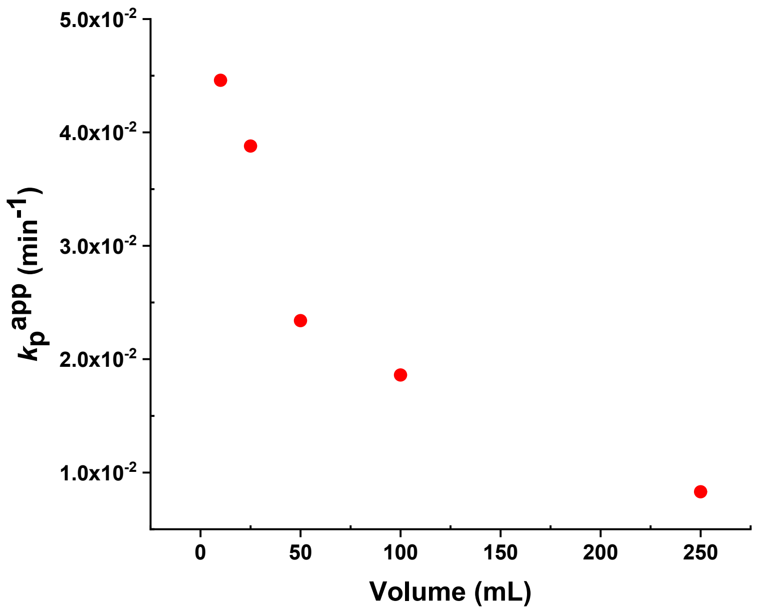

Figure S10. The relationship between k_p^{app} and volume of the polymerization mixture for SP-RAFT polymerization.

Expanding monomer scope of photo-RAFT polymerization

Synthesis of N-oxide-N,N-dimethylaminoethyl methacrylate (ODMA)

Tertiary amines oxide methacrylate was synthesized according to the previous publication with minor modifications.[31] Briefly, the solution of 3-chloroperbenzoic acid (6.5 g, 38 mmol) in dichloromethane (DCM) was added dropwise to the solution of DMAEMA (4.0 g, 25.3 mmol) in an ice bath. After 3 h of stirring at room temperature, the reaction was quenched by addition of sodium thiosulphate. The mixture was passed through a column filled with neutral alumina. The rotary evaporation of solvent yielded the final product, a yellow oil (yield: 52%).

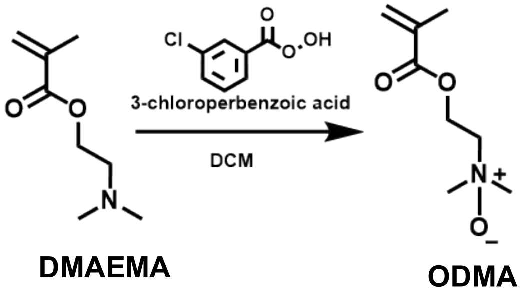

Scheme S1. Reaction scheme to synthesize ODMA.

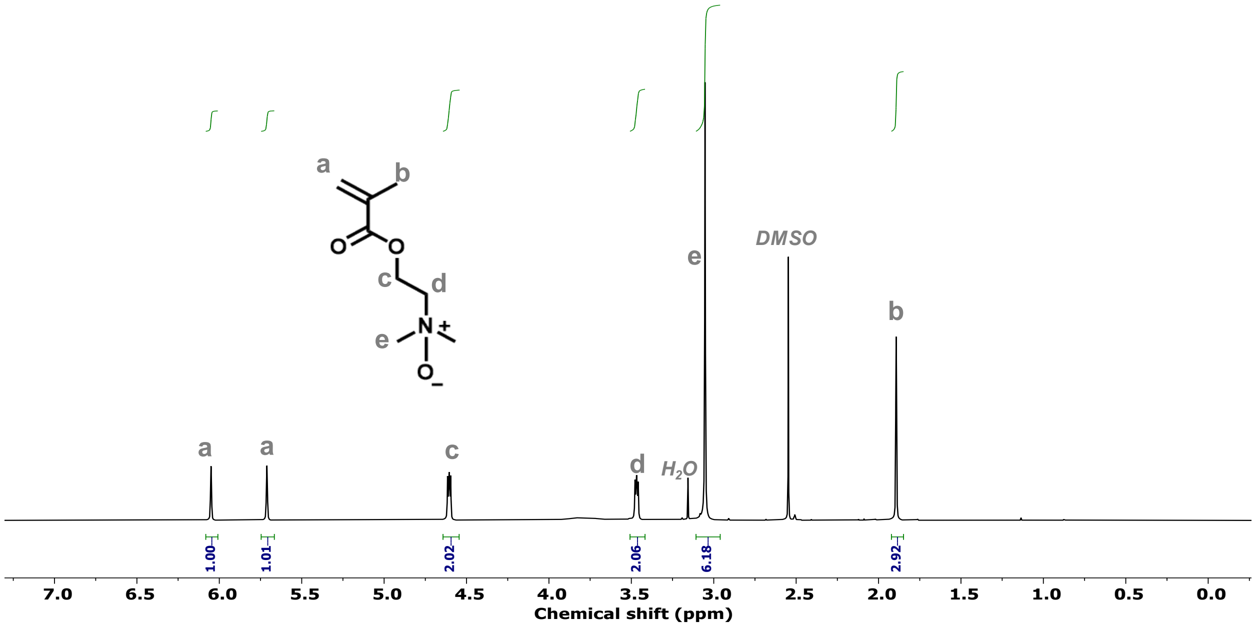

Figure S11. ^{1}H NMR spectrum of ODMA in DMSO-d_6.

Synthesis of 2-(methylsulfinyl)ethyl methacrylate (MSEMA)

A round bottom flask (250 mL) was charged with a stir bar, MAA (10.3 g, 0.12 mol), EDC (24 g, 0.12 mol), 2-(methylthio)ethanol (10 g, 0.11 mol) and DCM (150 mL) and placed in an ice bath. When the temperature was around 0 °C, DMAP (2.0 g, 16 mmol) was added dropwise into the reaction solution. The reaction was carried out for 24 h at 0 °C. The product was isolated by three extractions with 1 M HCl solution; the organic phase was dried with $MgSO_4$ and filtered through neutral alumina. DCM was removed by blowing air over the surface overnight.

The purified product was directly used for the oxidation with H_2O_2 (1.4 equiv.) which was added dropwise. The reaction mixture was left under stirring overnight. The product was isolated by performing three extractions with DCM. The organic phase was treated with a small amount of manganese dioxide (MnO_2) to completely remove the peroxide and $MgSO_4$ to remove any trace of water. After filtration, DCM was removed by air blowing overnight dried a vacuum desiccator for 24 h.

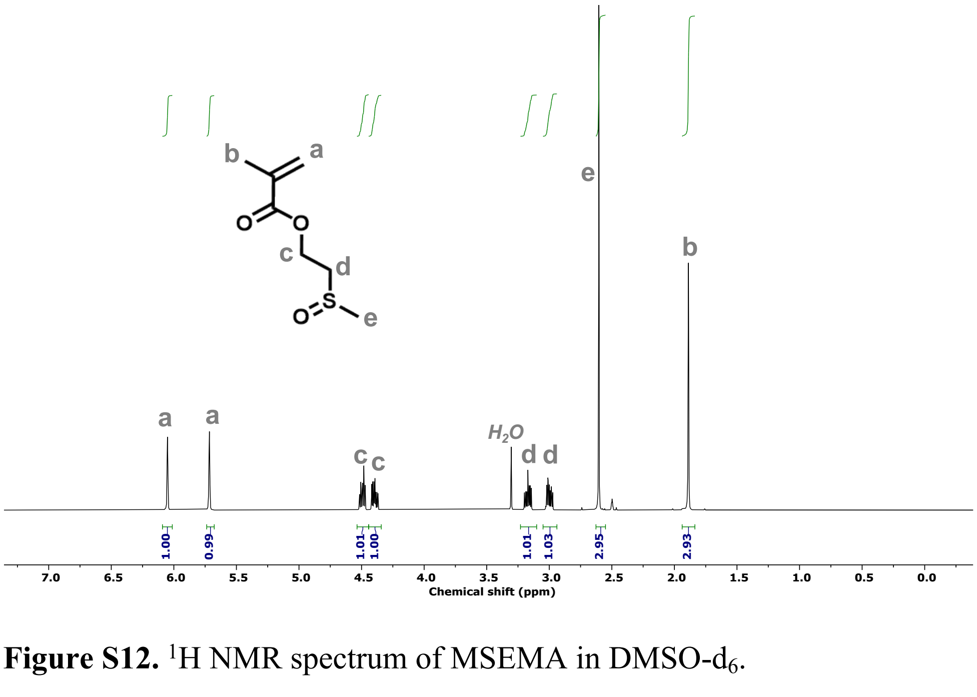

Figure S12. ^{1}H NMR spectrum of MSEMA in DMSO-d_6.

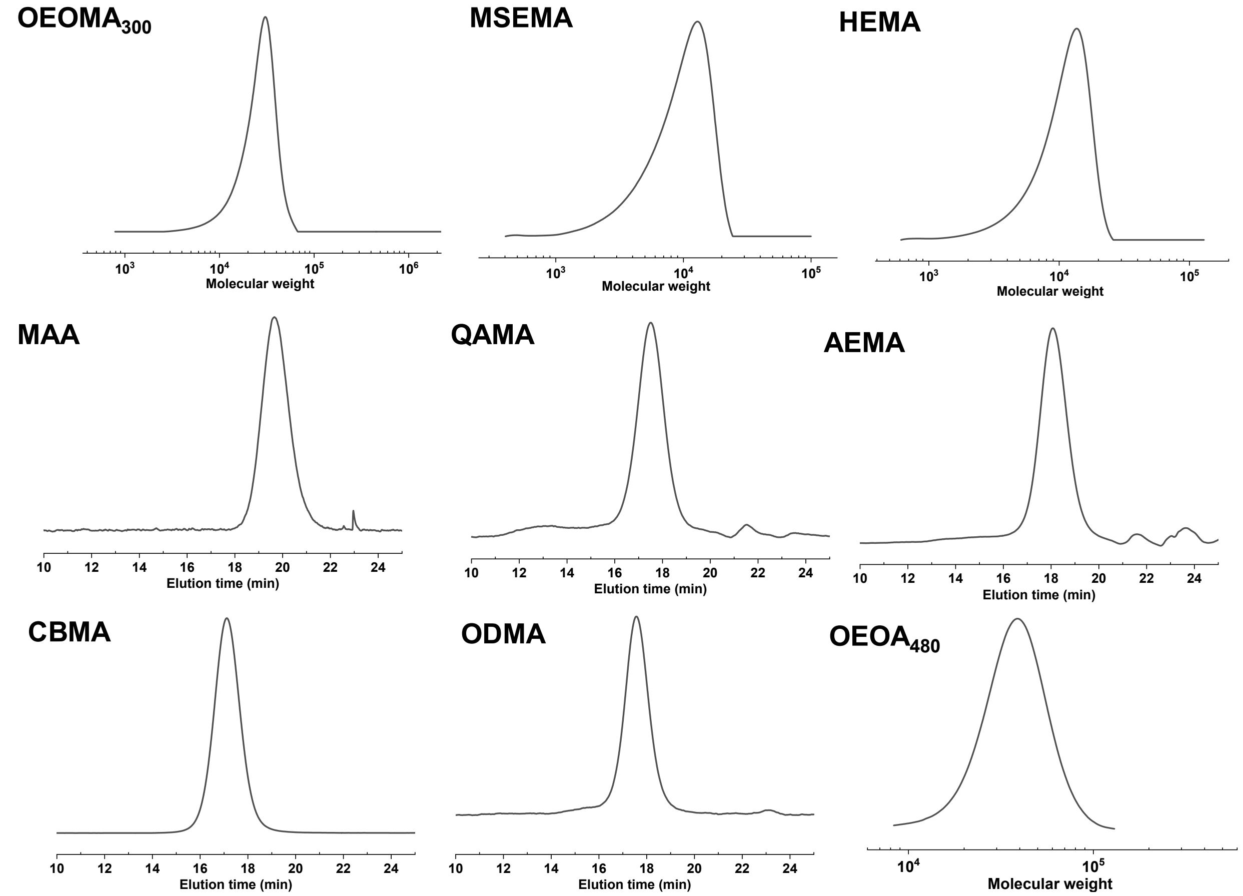

Figure S13. SEC traces of polymers synthesized by photo-RAFT polymerization with SP of different hydrophilic monomers.

Table S4. Photo-RAFT polymerization of $OEOA_{480}$

Entry	PAD	Light (nm)	$[OEOA_{480}]_0$ (mM)	Time (h)	^{b}Conv (%)	$M_{n,th}$	$^{c}M_{n,app}$	$^{c}Đ$
1	SP	370	300	1	99	47,800	33,800	1.18

$^{a)}$Reaction conditions: $[OEOA_{480}]_0$/[4-cyano-4-(((ethylthio)carbonothioyl)thio)pentanoic acid]$_0$/$[SP]_0$= 100/1/85 in water/DMSO (9/1 *v/v*) mixture, irradiated with UV light (370 nm, 6.5 mW/cm^2) in a glass vial. $^{b)}$Monomer conversion was determined by ^{1}H-NMR spectroscopy. $^{c)}$Molecular weight ($M_{n,app}$) and dispersity ($Đ$) were determined by SEC analysis (DMF as eluent) calibrated with poly(methyl methacrylate) standards.

Protein polymer hybrids (PPH) synthesis and characterization

Impact of UV light irradiation with/without SP on enzymatic activity of CT. A solution of native CT in PBS or 128 mM sodium pyruvate (6.8 mg CT/mL, 1 mL) was placed in a vial (2.0 mL) and was irradiated by light (395 nm, 165 mW per vial) at 16 °C for 30 min. The solution was diluted into 0.1 mg/mL with deionized water, and the effect of light irradiation on enzymatic activity of CT was obtained by comparison of Michaelis-Menten parameters against small peptide substrate, *N*-suc-Ala-Ala-Pro-Phe-pNA, in 100 mM sodium phosphate buffer (pH 8.0) at 37 °C. Briefly, in a cuvette, 0.1 M sodium phosphate buffer (900−990 µL, pH 8.0), substrate (0−80 µL, 6 mg/mL in DMSO), and enzyme 10 µL, 0.1 mg of chymotrypsin per 1 mL of 0.1 M sodium phosphate buffer (pH 8.0, 0.04 µM) were mixed. The rate of the hydrolysis was determined by recording the increase in absorbance at 412 nm for the first 20 s after mixing. k_{cat}, K_M and k_{cat}/K_M values were calculated using GraphPad Prism software when plotting substrate concentration versus initial hydrolysis velocity.

Table S5. Michaelis-Menten parameters of native CT after treatment with PBS (control), light, SP, and SP with light.

Sample	k_{cat} (s^{-1})	K_M (µM)	k_{cat}/K_M ($\mu M^{-1}\ s^{-1}$)
PBS	41.9 ± 0.9	80.8 ± 7.2	0.521 ± 0.047
UV light	37.6 ± 0.8	87.1 ± 7.7	0.433 ± 0.035
SP	43.9 ± 0.7	93.2 ± 6.0	0.471 ± 0.042
SP + light	30.5 ± 0.8	84.6 ± 9.4	0.360 ± 0.023

Synthesis of CPADB-NHS. To a solution of CPADB (420 mg, 1.5 mmol) in DCM (25 mL), DIC (320 µL, 2.0 mmol) and NHS (230 mg, 2.0 mmol) were added, and the mixture was stirred at 4 °C for 16 h (Scheme S2). The mixture was passed through the silica gel column, and then DCM was removed by rotary evaporator. CPADB-NHS was recrystallized in 2-propanol, Yield 480 mg (85

%). The chemical structure of CPADB-NHS was confirmed by ^{1}H NMR spectrum in $CDCl_3$ (Figure S14).

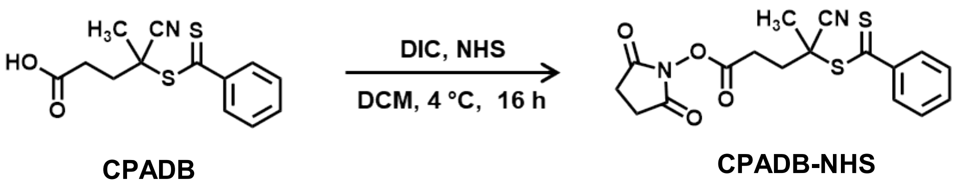

Scheme S2. Synthetic procedure for CPADB-NHS.

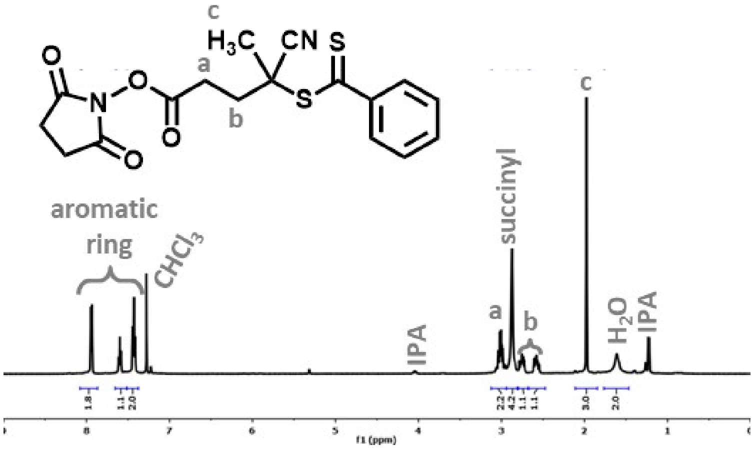

Figure S14. ^{1}H-NMR spectrum of CPADB-NHS in C*D*Cl_3.

Synthesis of CT-macro-RAFT agent (CT-CPADB). To a solution of CT (0.17 g, 6.8 μmol, 102 μmol amine group) in 100 mM sodium phosphate buffer (pH 8.0) in ice bath, a solution of CPADB-NHS (77 mg, 204 μmol) in DMSO (1.0 mL) was added dropwise. The mixture was stirred at 4 °C for 16 h. CT-macro-CPADB was purified by dialysis using 12-14 kDa molecular cutoff dialysis membrane in 25 mM sodium phosphate buffer (pH 7.0) and deionized water at 4 °C for 24 h. CT-macro-CPADB was obtained as light pink powder after lyophilization. The number of CPADB on CT was estimated by fluorescamine assay.

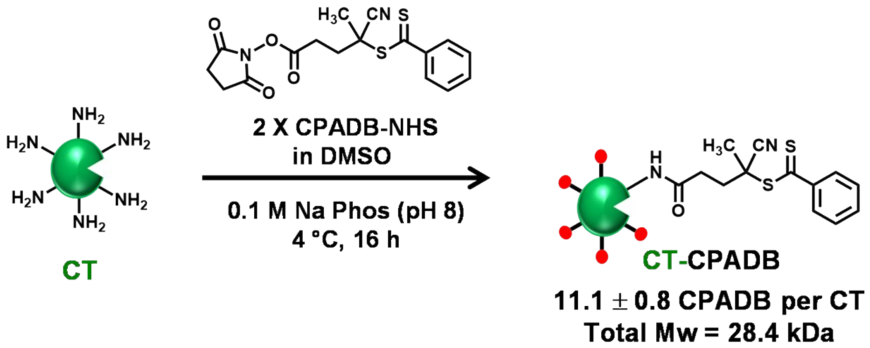

Scheme S3. Synthesis of CT-CPADB macro-RAFT agent.

Synthesis of protein-polymer hybrids (PPH). For preparing CT-POEOMA$_{500}$ PPH (entry 1 in Figure 7B), mixture of OEOMA$_{500}$ (150 mg, 0.30 mmol) and SP (7.1 mg, 0.065 mmol) were prepared in a volumetric flask (1 mL), followed by the addition of CT-CPADB (7.7 mg, 0.27 µmol). The volumetric flask (1 mL) was then topped up with ice-cold water. The final concentrations were OEOMA$_{500}$ (300 mM), CT-CPADB (0.27 mM), and SP (128 mM).

For preparing CT-PCBMA PPH (entry 2 in Figure 7B), mixture of CBMA (69 mg, 0.30 mmol) and SP (14.1 mg, 0.13 mmol) were mixed in a volumetric flask (1 mL), followed by the addition of CT-CPADB (7.7 mg, 0.27 µmol). The volumetric flask was then topped up with ice-cold water. The final concentrations were CBMA (300 mM), CT-CPADB (0.27 mM), and SP (128 mM).

For preparing CT-PCBMA PPH (entry 3 in Figure 7B), mixture of CBMA (69 mg, 0.30 mmol) and SP (14.1 mg, 0.13 mmol) were mixed in a volumetric flask (1 mL), followed by the addition of CT-CPADB (15.3 mg, 0.54 µmol). The volumetric flask was then topped up to 1 mL with ice-cold water. The final concentrations were: CBMA (300 mM), CT-CPADB (0.54 mM), and SP (128 mM).

The mixtures were then vortexed for 3 minutes, transferred to an open-to-air HPLC vial (2 mL), and was placed in the Lumidox® photoreactor equipped with violet light LEDs (395 nm, 165 mW/cm^2) and a cooling system (temperature during polymerization: 15–18 °C). The polymerization was stopped after 0.5 h by turning the light off. For SEC analysis, the polymerization solution was purified by dialysis using a 15 KDa molecular weight cut-off

dialysis tube in deionized water and then characterized by aqueous SEC equipped with MALS detector.

PPH kinetic assay. N-Succinyl-Ala-Ala-Pro-Phe-pNA (0 to 80 µL of 6.0 mM in DMSO) was added to sodium phosphate buffer (988 to 910 µL of 100 mM, pH 8.0). Native CT or conjugate solution (10 µL of 4.0 µM of CT) was added to the substrate solution. The initial rate of hydrolysis of the peptide substrate was monitored by recording the increase in absorption at 412 nm using an UV−vis spectrometer. The Michaelis−Menten parameters (k_{cat}, K_M, and k_{cat}/K_M) were determined by nonlinear curve fitting (equation for Michaelis−Menten parameters) of plots of initial rate versus substrate concentration using the Enzfitter software.

Table S6. Michaelis-Menten parameters of native, CT-CPADB and CT-polymer hybrids.

Sample	k_{cat} (s^{-1})	K_M (µM)	k_{cat}/K_M ($\mu M^{-1}\ s^{-1}$)
native CT	41.9 ± 0.9	80.8 ± 7.2	0.521 ± 0.047
CT-CPADB	3.6 ± 0.2	82.5 ± 12.9	0.044 ± 0.007
CT-PCBMA (DP = 50)	13.9 ± 0.3	67.3 ± 6.4	0.207 ± 0.020
CT-PCBMA (DP = 100)	14.8 ± 0.4	68.1 ± 7.3	0.217 ± 0.024
CT-PCBMA	23.1 ± 0.6	137.8 ± 11.6	0.168 ± 0.015

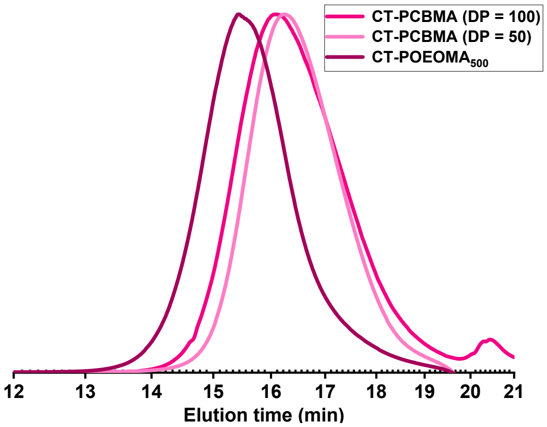

Figure S15. SEC traces (RI) of $POEOMA_{500}$- and PCBMA-CT hybrids.

References

(1) Chapman, R.; Gormley, A. J.; Herpoldt, K.-L.; Stevens, M. M. Highly Controlled Open Vessel RAFT Polymerizations by Enzyme Degassing. *Macromolecules* **2014**, *47* (24), 8541-8547.
(2) Gormley, A. J.; Chapman, R.; Stevens, M. M. Polymerization Amplified Detection for Nanoparticle-Based Biosensing. *Nano Letters* **2014**, *14* (11), 6368-6373.
(3) Zhang, B.; Wang, X.; Zhu, A.; Ma, K.; Lv, Y.; Wang, X.; An, Z. Enzyme-Initiated Reversible Addition–Fragmentation Chain Transfer Polymerization. *Macromolecules* **2015**, *48* (21), 7792-7802.
(4) Tan, J.; Liu, D.; Bai, Y.; Huang, C.; Li, X.; He, J.; Xu, Q.; Zhang, L. Enzyme-Assisted Photoinitiated Polymerization-Induced Self-Assembly: An Oxygen-Tolerant Method for Preparing Block Copolymer Nano-Objects in Open Vessels and Multiwell Plates. *Macromolecules* **2017**, *50* (15), 5798-5806.
(5) Liu, Z.; Lv, Y.; An, Z. Enzymatic Cascade Catalysis for the Synthesis of Multiblock and Ultrahigh-Molecular-Weight Polymers with Oxygen Tolerance. *Angewandte Chemie International Edition* **2017**, *56* (44), 13852-13856.
(6) Schneiderman, D. K.; Ting, J. M.; Purchel, A. A.; Miranda, R., Jr.; Tirrell, M. V.; Reineke, T. M.; Rowan, S. J. Open-to-Air RAFT Polymerization in Complex Solvents: From Whisky to Fermentation Broth. *ACS Macro Letters* **2018**, *7* (4), 406-411.
(7) Nothling, M. D.; McKenzie, T. G.; Eastland, I. A.; Chien, H.-C.; Collins, J.; Meyer, A. S.; Qiao, G. G. Self-deoxygenating glassware. *Chemical Communications* **2019**, *55* (59), 8544-8547.
(8) Lückerath, T.; Koynov, K.; Loescher, S.; Whitfield, C. J.; Nuhn, L.; Walther, A.; Barner-Kowollik, C.; Ng, D. Y. W.; Weil, T. DNA–Polymer Nanostructures by RAFT Polymerization and Polymerization-Induced Self-Assembly. *Angewandte Chemie International Edition* **2020**, *59* (36), 15474-15479.
(9) Bennett, M. R.; Moloney, C.; Catrambone, F.; Turco, F.; Myers, B.; Kovacs, K.; Hill, P. J.; Alexander, C.; Rawson, F. J.; Gurnani, P. Oxygen-Tolerant RAFT Polymerization Initiated by Living Bacteria. *ACS Macro Letters* **2022**, *11* (8), 954-960.
(10) Li, R.; Zhang, S.; Li, Q.; Qiao, G. G.; An, Z. An Atom-Economic Enzymatic Cascade Catalysis for High-Throughput RAFT Synthesis of Ultrahigh Molecular Weight Polymers. *Angewandte Chemie International Edition* **2022**, *61* (46), e202213396.
(11) Gody, G.; Barbey, R.; Danial, M.; Perrier, S. Ultrafast RAFT polymerization: multiblock copolymers within minutes. *Polymer Chemistry* **2015**, *6* (9), 1502-1511.
(12) Wu, H.; Yang, L.; Tao, L. Polymer synthesis by mimicking nature's strategy: the combination of ultra-fast RAFT and the Biginelli reaction. *Polymer Chemistry* **2017**, *8* (37), 5679-5687.
(13) Cosson, S.; Danial, M.; Saint-Amans, J. R.; Cooper-White, J. J. Accelerated Combinatorial High Throughput Star Polymer Synthesis via a Rapid One-Pot Sequential Aqueous RAFT (rosa-RAFT) Polymerization Scheme. *Macromolecular Rapid Communications* **2017**, *38* (8), 1600780.
(14) Shanmugam, S.; Xu, J.; Boyer, C. Aqueous RAFT Photopolymerization with Oxygen Tolerance. *Macromolecules* **2016**, *49* (24), 9345-9357.
(15) Wang, J.; Rivero, M.; Muñoz Bonilla, A.; Sanchez-Marcos, J.; Xue, W.; Chen, G.; Zhang, W.; Zhu, X. Natural RAFT Polymerization: Recyclable-Catalyst-Aided, Opened-to-Air, and Sunlight-Photolyzed RAFT Polymerizations. *ACS Macro Letters* **2016**, *5* (11), 1278-1282.

(16) Ren, K.; Perez-Mercader, J. Thermoresponsive gels directly obtained via visible light-mediated polymerization-induced self-assembly with oxygen tolerance. *Polymer Chemistry* **2017**, *8* (23), 3548-3552.
(17) Yeow, J.; Chapman, R.; Xu, J.; Boyer, C. Oxygen tolerant photopolymerization for ultralow volumes. *Polymer Chemistry* **2017**, *8* (34), 5012-5022.
(18) Yu, L.; Wei, Y.; Tu, Y.; Lin, S.; Huang, Z.; Hu, J.; Chen, Y.; Qiao, H.; Zou, W. An oxygen-tolerant photo-induced metal-free reversible addition-fragmentation chain transfer polymerization. *Journal of Polymer Science Part A: Polymer Chemistry* **2018**, *56* (21), 2437-2444.
(19) Wu, Z.; Fang, W.; Wu, C.; Corrigan, N.; Zhang, T.; Xu, S.; Boyer, C. An aqueous photo-controlled polymerization under NIR wavelengths: synthesis of polymeric nanoparticles through thick barriers. *Chemical Science* **2022**, *13* (39), 11519-11532, 10.1039/D2SC03952D.
(20) Lee, Y.; Kwon, Y.; Kim, Y.; Yu, C.; Feng, S.; Park, J.; Doh, J.; Wannemacher, R.; Koo, B.; Gierschner, J.; Kwon, M. S. A Water-Soluble Organic Photocatalyst Discovered for Highly Efficient Additive-Free Visible-Light-Driven Grafting of Polymers from Proteins at Ambient and Aqueous Environments. *Advanced Materials* **2022**, *34* (14), 2108446.
(21) Li, Z.; Lian, Q.; Xu, Y.; Zhang, Y.; Zhang, P.; Geng, J. Aggregation-Induced Emission Luminogen Catalyzed Photocontrolled Reversible Addition–Fragmentation Chain Transfer Polymerization in an Aqueous Environment. *Macromolecules* **2022**, *55* (7), 2904-2910.
(22) Li, X.; Huang, Y.; Wei, W. F.; Guo, W. L.; Luo, Z.; Xu, J.; Cai, T. Metalloporphyrin-anchored 2D MOF nanosheets as highly accessible heterogeneous photocatalysts towards cytocompatible living radical polymerization. *Chemical Engineering Journal* **2022**, *434*, 134692.
(23) Yang, H.; Lu, Z.; Fu, X.; Li, Q.; Xiao, L.; Zhao, Y.; Hou, L. Visible light-triggered PET-RAFT polymerization by heterogeneous 2D porphyrin-based COF photocatalyst under aqueous condition. *European Polymer Journal* **2022**, *173*, 111306.
(24) Yu, C.; Song, J.; Kim, T. I.; Lee, Y.; Kwon, Y.; Kim, J.; Park, J.; Choi, J.; Doh, J.; Min, S. K.; et al. Silver Sulfide Nanocrystals as a Biocompatible and Full-Spectrum Photocatalyst for Efficient Light-Driven Polymerization under Aqueous and Ambient Conditions. *ACS Catalysis* **2023**, *13* (1), 665-680.
(25) Fan, J.; Li, C.; Guo, X.; Deng, Y. Methylene blue sensitized photo Fenton-like reaction for rapid RAFT polymerization in aqueous solution. *Polymer Chemistry* **2023**, *14* (6), 773-783.
(26) Lu, Z.; Yang, H.; Zhao, R.; Zhao, Y.; Xiao, L.; Hou, L. Ligand regulation strategy of COF-based photocatalyst for ROS-mediated RAFT polymerization. *Polymer Chemistry* **2023**, *14* (19), 2291-2298.
(27) Deng, Y.; Li, C.; Fan, J.; Guo, X. Photo Fenton RAFT Polymerization of (Meth)Acrylates in DMSO Sensitized by Methylene Blue. *Macromolecular Rapid Communications* **2023**, *44* (19), 2300258.
(28) Moini Jazani, A.; Rawls, C.; Matyjaszewski, K. Photo-RDRP for everyone: Smartphone light-induced oxygen-tolerant reversible deactivation radical polymerization. *European Polymer Journal* **2024**, *202*, 112631.
(29) Guzman, M. I.; Eugene, A. J. Aqueous Photochemistry of 2-Oxocarboxylic Acids: Evidence, Mechanisms, and Atmospheric Impact. *Molecules* **2021**, *26* (17), 5278.
(30) Thum, M. D.; Wolf, S.; Falvey, D. E. State-Dependent Photochemical and Photophysical Behavior of Dithiolate Ester and Trithiocarbonate Reversible Addition–Fragmentation Chain Transfer Polymerization Agents. *The Journal of Physical Chemistry A* **2020**, *124* (21), 4211-4222.

(31) Zhang, L.; Sun, J.; Huang, W.; Zhang, S.; Deng, X.; Gao, W. Hypoxia-Triggered Bioreduction of Poly(N-oxide)–Drug Conjugates Enhances Tumor Penetration and Antitumor Efficacy. *Journal of the American Chemical Society* **2023**, *145* (3), 1707-1713.
